# The transcription factor LEF1 interacts with NFIX and switches isoforms during adult hippocampal neural stem cell quiescence

**DOI:** 10.3389/fcell.2022.912319

**Published:** 2022-07-22

**Authors:** Laura García-Corzo, Isabel Calatayud-Baselga, Lucía Casares-Crespo, Carlos Mora-Martínez, Juan Julián Escribano-Saiz, Rafael Hortigüela, Andrea Asenjo-Martínez, Antonio Jordán-Pla, Stefano Ercoli, Nuria Flames, Victoria López-Alonso, Marçal Vilar, Helena Mira

**Affiliations:** ^1^ Instituto de Biomedicina de Valencia, Consejo Superior de Investigaciones Científicas (IBV-CSIC), València, Spain; ^2^ Evo-devo Helsinki Community, Centre of Excellence in Experimental and Computational Developmental Biology, Institute of Biotechnology, University of Helsinki, Helsinki, Finland; ^3^ Instituto de Salud Carlos III (ISCIII), Majadahonda, Spain

**Keywords:** neural stem cells, adult hippocampal neurogenesis, quiescence, Wnt signalling, LEF1, NFIX, alternative splicing

## Abstract

Stem cells in adult mammalian tissues are held in a reversible resting state, known as quiescence, for prolonged periods of time. Recent studies have greatly increased our understanding of the epigenetic and transcriptional landscapes that underlie stem cell quiescence. However, the transcription factor code that actively maintains the quiescence program remains poorly defined. Similarly, alternative splicing events affecting transcription factors in stem cell quiescence have been overlooked. Here we show that the transcription factor T-cell factor/lymphoid enhancer factor LEF1, a central player in canonical β-catenin-dependent Wnt signalling, undergoes alternative splicing and switches isoforms in quiescent neural stem cells. We found that active β-catenin and its partner LEF1 accumulated in quiescent hippocampal neural stem and progenitor cell (Q-NSPC) cultures. Accordingly, Q-NSPCs showed enhanced TCF/LEF1-driven transcription and a basal Wnt activity that conferred a functional advantage to the cultured cells in a Wnt-dependent assay. At a mechanistic level, we found a fine regulation of *Lef1* gene expression. The coordinate upregulation of *Lef1* transcription and retention of alternative spliced exon 6 (E6) led to the accumulation of a full-length protein isoform (LEF1-FL) that displayed increased stability in the quiescent state. Prospectively isolated GLAST + cells from the postnatal hippocampus also underwent E6 retention at the time quiescence is established *in vivo*. Interestingly, LEF1 motif was enriched in quiescence-associated enhancers of genes upregulated in Q-NSPCs and quiescence-related NFIX transcription factor motifs flanked the LEF1 binding sites. We further show that LEF1 interacts with NFIX and identify putative LEF1/NFIX targets. Together, our results uncover an unexpected role for LEF1 in gene regulation in quiescent NSPCs, and highlight alternative splicing as a post-transcriptional regulatory mechanism in the transition from stem cell activation to quiescence.

## Introduction

Tissue homeostasis and repair in a variety of mammalian organs and tissues relies on the persistence of dedicated somatic stem cell reservoirs (Muñoz-Cánoves and Huch, 2018; van Velthoven and Rando, 2019). Throughout adulthood, these resident stem cells are kept predominantly in a reversible resting state known as quiescence. Recent studies suggest that quiescence is more than just a passive latency condition developed to protect stem cells from the drawbacks of hyperproliferation, such as stem cell depletion and DNA damage. Despite being metabolically less active than their proliferating counterparts, at least in some systems, quiescent stem cells seem to remain in a flexible state that allows them to respond quickly to changes in their environment (van Velthoven and Rando, 2019). Such a state requires a fine regulation of gene expression. Recent research also shows the existence of defined levels or degrees of quiescence among the adult stem cell populations. In certain niches or in specific circumstances, pools of quiescent stem cells have been found in a shallow quiescent state, primed or ready for re-activation and differentiation (Llorens-Bobadilla et al., 2015; Rodgers et al., 2017; Harris et al., 2021; Marqués-Torrejón et al., 2021).

Within the brain, a prominent quiescent neural stem cell (NSC) reservoir is maintained in the subgranular zone of the hippocampal dentate gyrus, a region immediately adjacent to a densely packed layer of glutamatergic granule neurons (Moss et al., 2016). The development of this stem cell niche ends during the postnatal period, coinciding with the transition of NSCs from the proliferative state into the definitive quiescent state several weeks after birth (Berg et al., 2019; Morales and Mira, 2019). Thereafter, only a minor fraction of the adult hippocampal NSCs becomes active and engages in a neurogenic cascade that leads to the production of new functional granule neurons involved in learning and memory tasks (Gonçalves et al., 2016; Morales and Mira, 2019).

We previously reported that stem cell quiescence in the adult hippocampus is maintained by BMP4 signalling acting downstream of the BMPR1A receptor expressed by radial glia-like NSCs (Mira et al., 2010), a finding widely corroborated thereafter (Martynoga et al., 2013; Knobloch et al., 2017; Sueda et al., 2019). The equilibrium between NSC quiescence and activation leading to productive neurogenesis depends on the interplay of BMP signalling and a variety of other local hippocampal niche signals, the Wnt family being of utmost importance (Lie et al., 2005; Michaelidis and Lie, 2008; Varela-Nallar and Inestrosa, 2013; Choe et al., 2015; Armenteros et al., 2018). Two branches of the Wnt signalling pathway influence adult neurogenesis progression in the hippocampus. Canonical signalling affects both the early and the late stages of the neurogenic progression and dictates proliferation, neuronal specification, dendritic growth and spine formation (Lie et al., 2005; Kuwabara et al., 2009; Qu et al., 2013; Heppt et al., 2020), while non-canonical signalling influences maturation (Schafer et al., 2015; Ortiz-Matamoros and Arias, 2019; Arredondo et al., 2020). In the canonical pathway, secreted Wnt ligands bind to a complex of frizzled (FZD) receptors and co-receptors, phosphorylating Dishevelled that in turn recruits the beta-catenin (β-catenin) destruction complex formed by glycogen synthase kinase 3 beta (GSK3B), casein kinase 1α (CK1α), APC (Adenomatosis Polyposis Coli) and Axin among other proteins, allowing β-catenin accumulation and nuclear translocation (Clevers and Nusse, 2012). Thereafter, β-catenin binds to T cell factor/Lymphoid enhancer factor 1 (TCF/LEF1) transcription factors in order to regulate a panoply of Wnt target genes, including Wnt signalling components such as *Lef1* and *Axin2* as part of a series of feedback loops (Hovanes et al., 2001; Lustig et al., 2002; Clevers et al., 2014).

Interfering with Wnt signaling in the hippocampus reduces the number of proliferating precursors and the percentage of the cells that engage in neuronal differentiation (Lie et al., 2005; Kuwabara et al., 2009; Qu et al., 2013; Heppt et al., 2020). *In vitro*, canonical Wnt signalling enhances proliferation through the binding of β-catenin to TCF/LEF1 sites at the Cyclin D1 promoter while it promotes neuronal specification and differentiation through the binding to the promoter of the proneural transcription factors Neurogenin 2 and NeuroD1 (Lie et al., 2005; Kuwabara et al., 2009; Qu et al., 2013; Heppt et al., 2020). Among all TCF/LEF factors, LEF1 is key for the proper development of the hippocampal dentate gyrus (Galceran et al., 2000; Zhou et al., 2004). Compared to other TCF/LEF1 family members that are more widely distributed in the brain, early in development LEF1 is selectively expressed in the dentate neuroepithelium (Galceran et al., 2000; Zhou et al., 2004), the germinal source of the dentate granule neurons that is also the origin of adult hippocampal NSCs (Berg et al., 2019; Morales and Mira, 2019). Accordingly, LEF1-deficient embryos specifically fail to generate dentate gyrus granule neurons and show a severe loss of dentate precursor cells (Galceran et al., 2000; Zhou et al., 2004). In contrast to the well-established roles of LEF1 during embryonic development, the potential function of LEF1 in the quiescent NSC reservoir during adulthood remains poorly explored (Lie et al., 2005; Kuwabara et al., 2009; Qu et al., 2013; Heppt et al., 2020; Austin et al., 2021).

Although great progress has been made in identifying the temporal sequence of transcription factors that regulates the activation of adult hippocampal NSCs (Andersen et al., 2014; Urbán et al., 2016; Blomfield et al., 2019; Li et al., 2022) and the progression along the neurogenic cascade (Ozen et al., 2007; Gao et al., 2009; Haslinger et al., 2009; Roybon et al., 2009; Hodge et al., 2012; Matsuda et al., 2012; Mu et al., 2012; Amador-Arjona et al., 2015), little is known regarding the combination or code of transcription factors that actively maintain the quiescent state. The quiescence programme downstream of BMP signalling is presumably regulated by the BMP-dependent canonical SMAD1 (Mira et al., 2010), REST (Mukherjee et al., 2016) and NFIX transcription factors (Martynoga et al., 2013) while activation largely depends on ASCL1 proneural transcription factor and its modulators (Andersen et al., 2014; Urbán et al., 2016; Blomfield et al., 2019; Sueda et al., 2019; Li et al., 2022). Whether other transcription factors play a role in regulating specific aspects of the physiology of quiescent NSCs, such as metabolic rewiring or priming, remains to be elucidated.

We herein investigate the molecular program that regulates quiescence employing adult hippocampal neural stem and progenitor cells (NSPCs) cultures reversibly arrested by the quiescence-promoting signal BMP4 in the presence of the mitogen FGF2 (Mira et al., 2010), a valuable model that simulates *in vitro* an induced neural stem cell quiescent state that has been recently suggested to mimic a primed-like quiescence (Marqués-Torrejón et al., 2021). We confirm previous data showing that entry into quiescence involves major changes in the transcriptional profile of the cultured cells that evidence a shift in metabolism and the upregulation of genes related to a variety of signalling pathways, including Wnt signalling components. This suggests that the quiescent NSPCs may be predisposed to sense and respond to changes in their local microenvironment. We demonstrate that quiescent NSPCs display higher basal levels of active β-catenin and TCF/LEF transcriptional activity compared to proliferating NSPCs and respond to canonical Wnt signalling. The increased TCF/LEF1/β-catenin signalling correlated with the accumulation of LEF1 at the mRNA and protein level. We also uncover a differential regulation of *Lef1* E6 alternative splicing during the transition from proliferation to quiescence that results in the accumulation of a highly stable LEF1 full-length (LEF1-FL) isoform in quiescent cells. This long isoform also accumulates *in vivo* during postnatal development at the time NSCs settle in the hippocampal niche as a quiescent reservoir. In addition, we identify active enhancers in quiescent NSPCs that are enriched in putative LEF1 DNA-binding sites, with NFY and NFI motifs in their flanking sequences, and show that LEF1 is able to physically interact with NFIX. Finally, our results identify several candidate LEF1 target genes upregulated in quiescent cells that warrant further investigation.

## Materials and methods

### Cell culture

For proliferation, quiescence and differentiation assays we used rat adult hippocampal Neural Stem and Progenitor Cells (NSPCs) (Palmer et al., 1997; Mira et al., 2010). For half-life experiments, cells were incubated with cycloheximide (CHx; 100 mg/ml; Sigma Aldrich) and lysed at different timepoints. HEK293T were cultured in Dulbecco’s Modified Eagle’s Medium (DMEM, Corning) containing 10% fetal bovine serum (FBS, Corning), 2 mM L-Glutamine (Lonza) and 100 U/mL of Penicillin-Streptomycin antibiotic (Lonza) and were transfected with plasmids as described in Supplementary Material. Cells were cultured at 37°C in a humidified atmosphere with 5% of CO2.

### Microarray and RNAseq

For the transcriptomic analysis employing microarrays, total RNA was extracted from the NSPCs and cDNA was synthesized, fragmented, labelled and hybridized to the GeneChip Rat Gene 1.1 ST microarrays (Affymetrix). Data was deposited in the gene expression omnibus GEO dataset (GSE158658). Single-cell RNAseq data from hippocampal Nestin-GFP NSCs were adapted from Shin et al. (2015) (see the number of regulated genes per pathway in Figure 6). Additional information can be found in Supplementary Material.

### RT-PCR and estimation of exon retention

RNA was extracted from Q- and A-NSPCs at 4 DIV. Gene expression was determined by quantitative PCR (qPCR) using TB Green Premix EX Taq (Takara). Data were analysed according to the 2^−ΔΔCt^ method (Livak and Schmittgen, 2001). Forward and reverse primers for each gene are shown in Supplementary Material. *Sdha* was used for normalization. Efficiency of splicing was calculated by standard RT-PCR. Products were resolved in agarose gel electrophoresis and gel images were analysed with ImageJ Fiji software. Percentage of exon retention was calculated as described in Supplementary Material.

### Western blot and immunoprecipitation

Cells were lysed and fractionated by SDS-PAGE. Membranes were incubated with primary and peroxidase-conjugated secondary antibodies as described in Supplementary Material. ECL signals were analysed with ImageJ Fiji software and normalised against the intensity of β-actin. For the LEF1-FL detection with anti-LEF1-E6 and for NFIX-HA and LEF1-GFP co-immunoprecipitation assays, membranes were imaged by infrared imaging system with Odyssey^®^ (Li-cor Biosciences) and were analysed using the Image Studio Lite (Li-cor) program. Co-immunoprecipitation was performed as described in Supplementary Material.

### Wnt reporter assays

For the 7xTCF-eGFP-SV40-mCherry (p7GC) assay, NSPCs were transduced with the p7GC lentiviral vector (Addgene #24304). eGFP gene expression was normalised to mCherry expression by RT-qPCR. TOPFlash Luciferase assays were performed in Neuro2A cells as described in Supplementary Material.

### GLAST^+^ cell isolation

Briefly, hippocampi were dissected from C57BL/6JRccHsd P3-P21 mice and were dissociated employing the gentleMACS Octo Dissociator (Miltenyi Biotec). Magnetically labelled GLAST-positive cells were separated by MACS with Anti-GLAST (ACSA-1) MicroBead Kit (Miltenyi Biotec) and were subsequently analysed (see Supplementary Material).

### Motif analysis in enhancers

DNA sequences for previously reported NSPC enhancers (Martynoga et al., 2013; Knobloch et al., 2017; Sueda et al., 2019) were retrieved from the masked version of the *Mus musculus* MGSCv37 (mm9) genome assembly. Raw count matrices for LEF, SOX2, NFIX, ASCL1, and ASCL2 were downloaded from the JASPAR database and transformed into position weight matrices (PWMs). PWMs were aligned to masked sequences using the match PWM function from the Biostrings package. Only the maximally scoring motif was considered. To test for preferential LEF motif localization, we used the CentriMo tool of the MEME suite using default parameters. Motifs enriched in the vicinity of LEF putative binding sites were retrieved with the DREME tool of the MEME suite. Enriched motifs were then fed to the TOMTOM program in order to find matches with known PWMs (see Supplementary Material for additional details and references).

To analyse LEF Motif presence in genes differentially regulated in quiescent vs. proliferative cells, we used RNA-seq and ChIP-seq data from (Martynoga et al., 2013). We considered that genes were up or down regulated if they had a *p*-value < 0.01. Gene-enhancer associations were taken from (Martynoga et al., 2013). Briefly, by crossing these data with our previous motif search, we built a 3 × 2 contingency matrix with upregulated, downregulated and not differentially regulated genes vs. presence and absence of LEF motif and applied a Chi-squared test (see Supplementary Material for additional details).

### Statistical analysis

For the statistical analysis, all data were analyzed with PRISM (GraphPad 8). The Shapiro-Wilk test was used to test for the normal distribution of data. When data were normally distributed, then the two-tailed unpaired or paired Student’s *t* test or one/two-way ANOVA was used. For data presented as a fold increase, the One-sample *t* test was employed. When data were not normally distributed, then the Mann-Whitney test was used. Data are presented as mean ± SEM or median ± interquartile range for normally and non-normally distributed data, respectively. *p*-values less than 0.05 were considered as significant (*p < 0.05, **p < 0.01, ***p < 0.001).

## Results

### Molecular signature and niche signal integration capacity of quiescent adult hippocampal neural stem and progenitor cells

**FIGURE 1:**
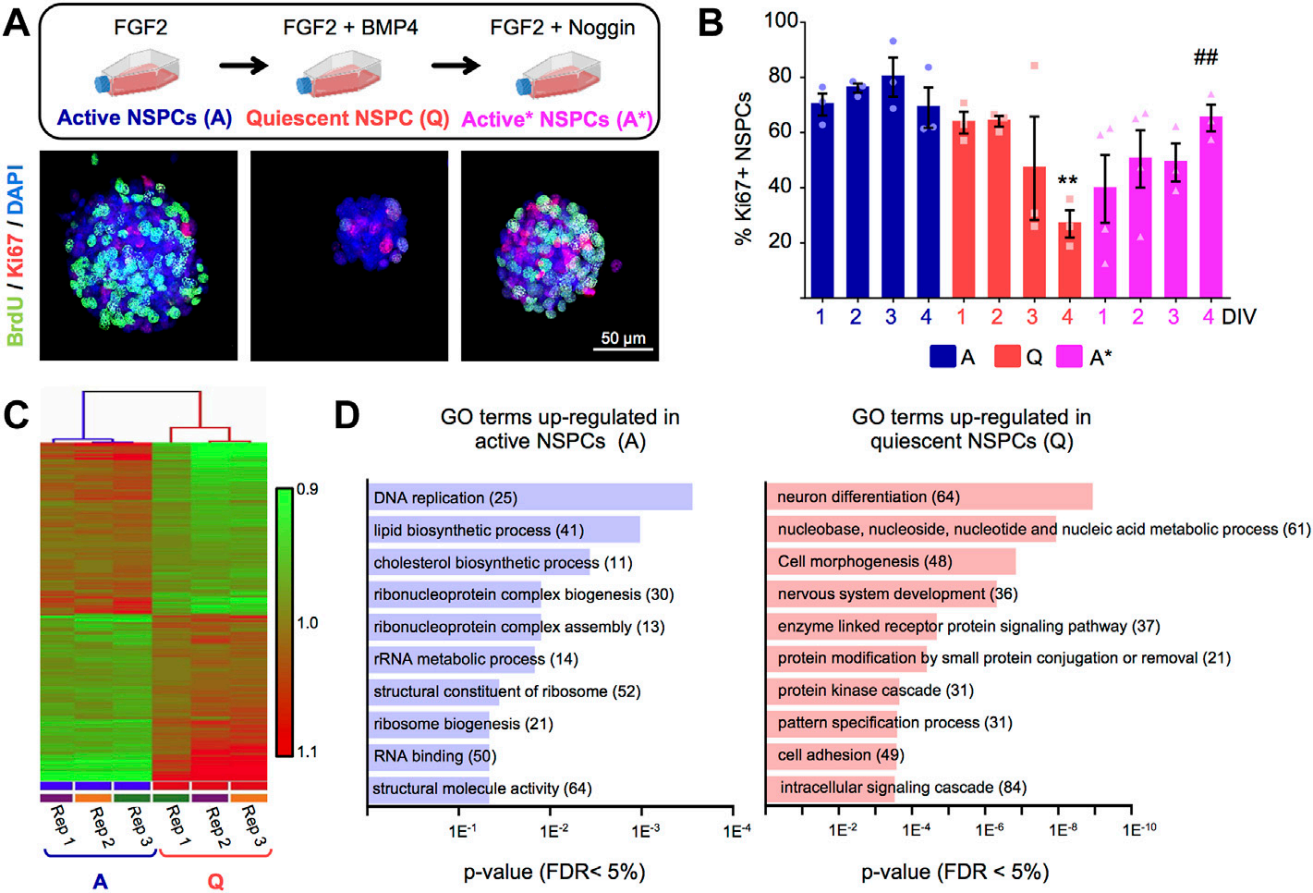
**(A)** *Upper panel*, *in vitro* experimental setup employed to induce the entrance and exit from quiescence of adult hippocampal neural stem and progenitor cells (NSPCs) grown as neurospheres, using FGF2+BMP4 and FGF2+Noggin, respectively. *Lower panel*, representative confocal images of active and quiescent NSPC neurospheres stained with BrdU and Ki67 markers. Quiescent NSPCs showed a reduction in the expression of both markers. **(B)** Quantification of the percentage of Ki67^+^ cells in active (A) and quiescent (Q) NSPCs and in quiescent NSPCs treated with Noggin to induce again activation (A*) (*n* ≥ 3, two-way ANOVA time *p*-value = 0.6 and treatment *p* < 0.01). **A-NSPC vs. Q-NSPC (4DIV), ## A*-NSPC (4DIV) vs. Q-NSPC (4DIV) (*p*-values in multiple *t*-test: <0.01 and <0.01, respectively). **(C)** Hierarchical clustering analysis of the genome-wide microarray data from Q-NSPC and A-NSPC (n = 3). **(D)** Gene ontology (GO) analysis using DAVID of the upregulated genes in A-NSPC and Q-NSPC (FDR <5%). Genes were classified by the category of biological processes. The number of genes involved in each process are shown between brackets. DIV, days *in vitro*. Data are presented as Mean ± SEM. *p < 0.05; **p < 0.01; ***p < 0.001. Scale in B: 50 μm. Flasks in (A) were retrieved from BioRender.com.

To model quiescence of adult hippocampal neural stem and progenitor cells (NSPCs) *in vitro*, we employed a previously described method (Mira et al., 2010; Sun et al., 2011). Briefly, we expanded the cells in culture as proliferating neurospheres in the presence of mitogenic stimulation (fibroblast growth factor 2, FGF2) and then supplemented the growth medium with BMP4 to efficiently induce the quiescent state (Figure 1A). We followed the dynamics of cell cycle exit by staining for the cell cycle marker Ki67 and confirmed that BMP4-treated cells progressively stopped dividing and entered quiescence (G_0_). At 4 days *in vitro* (DIV), we found a significant reduction in the percentage of Ki67^+^ cells (Figures 1A,B) and in the average size of the neurospheres (Supplementary Figure S1A,B). The cell cycle arrest was reversible, since adding the BMP antagonist Noggin to the medium allowed the NSPCs to re-enter the cell cycle and resume proliferation (Figures 1A,B). Similar results were observed when the proliferative activity of the cells was tracked through the incorporation of the thymidine analogue BrdU (Supplementary Figure S1D). Moreover, quiescent cells remained undifferentiated and maintained the expression of NSPCs markers such as Sox2 (Supplementary Figure S1C) as previously reported (Mira et al., 2010; Martynoga et al., 2013).

We next exploited this *in vitro* system to elucidate the molecular program underlying the quiescent state of adult hippocampal NSPCs. Global transcriptome analysis employing genome-wide microarrays was performed on quiescent cells (“Q-NSPCs”, 4DIV FGF2+BMP4 treated neurospheres) and on actively proliferating cells (“A-NSPCs”, 4DIV FGF2 treated neurospheres.) Hierarchical clustering analysis of the microarray data showed that Q-NSPC cultures clustered together and separately from A-NSPC cultures (Figure 1C), indicating that their transcriptional profile is clearly distinguishable. The Q- and A-NSPC samples were also segregated on the principal component analysis plot (PCA, Supplementary Figure S1E).

We found that 1,314 genes were significantly upregulated in quiescent NSPCs compared to active NSPCs while 1,023 genes were downregulated (*p* < 0.05; Supplementary Table S1; the Volcano Plot obtained for the working list is shown in Supplementary Figure S1F). Gene ontology (GO) analysis using DAVID (Database for Annotation, Visualization, and Integrated Discovery; http://david.abcc.ncifcrf.gov) showed that mRNAs upregulated in Q-NSPCs were mostly involved in developmental cell decisions (GO terms such as “nervous system development,” “cell morphogenesis,” “pattern specification process,” and “neuron differentiation”) and cellular signalling (GO terms: “intracellular signalling cascade,” “protein kinase cascade,” and “enzyme linked receptor protein signalling pathway”) (Figure 1D). In addition, pathway analysis with KEGG (Kyoto Encyclopedia of Genes and Genomes) revealed a shift in metabolism, i.e., genes related to fatty acid and protein degradation were enriched in Q-NSPCs (KEGG terms: “fatty acid metabolism,” “ubiquitin mediated proteolysis,” “lysosome”; Supplementary Table S2). Notably, the molecular signature of quiescent adult hippocampal NSPCs in culture closely resembled that of their *in vivo* counterparts, as reported in single-cell RNAseq studies (Shin et al., 2015) and was also similar to the signature of quiescent NSCs derived from ES cells treated with BMP4 (Martynoga et al., 2013). Conversely, upregulated gene categories in actively proliferating NSPCs were associated with ribosome biogenesis (e.g., GO terms: “structural constituent of ribosome,” “ribosome biogenesis”), cell cycle (“DNA replication”), RNA metabolism (“rRNA metabolic process,” “RNA binding”) and lipid metabolism (“lipid biosynthetic process,” “cholesterol biosynthetic process”) (Figure 1D). Gene categories related to these same processes were also enriched in A-NSPCs according to KEGG (KEGG terms: “ribosome,” “cell cycle,” “spliceosome”; Supplementary Table S2).

**FIGURE 2:**
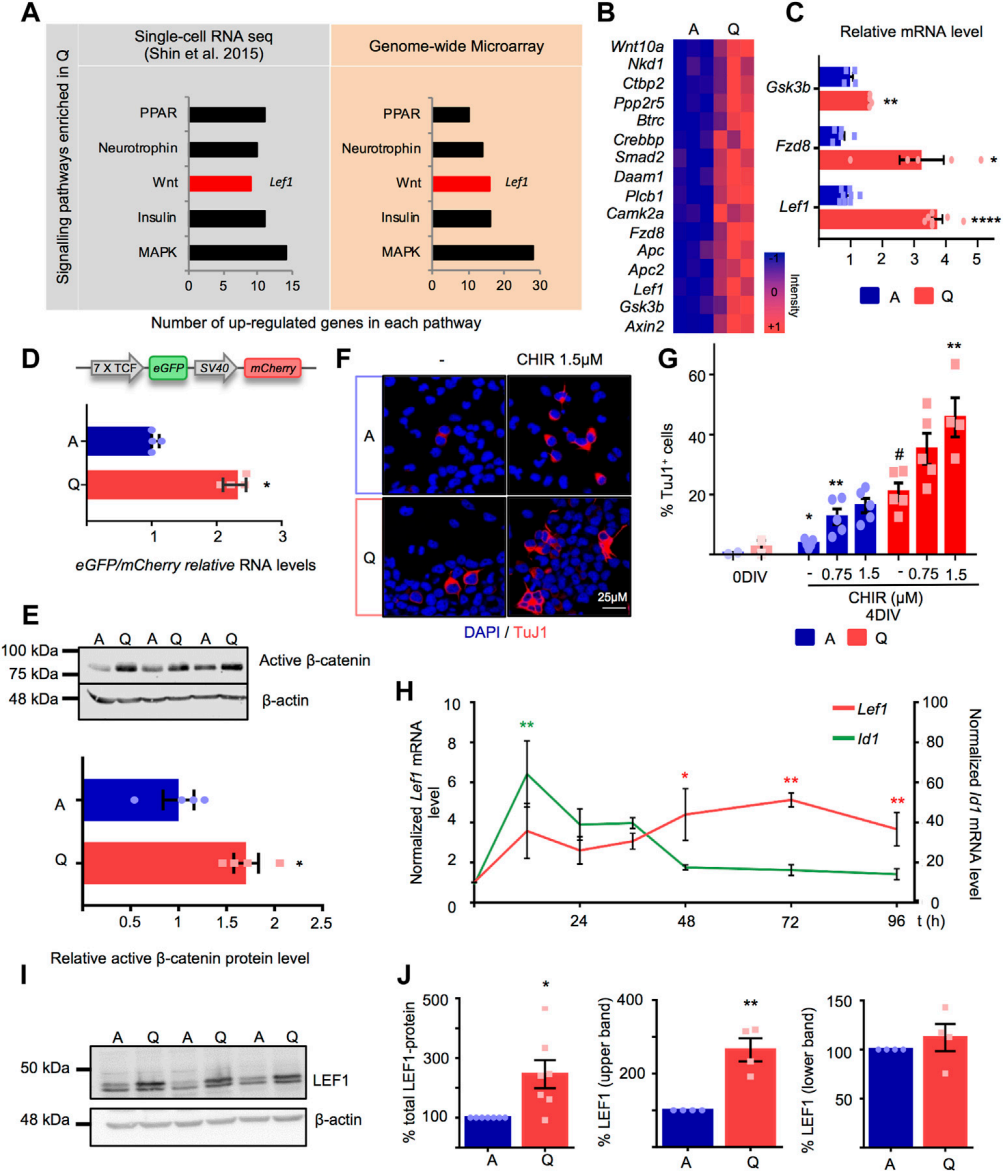
**(A)** Signalling pathways enriched in Q-NSPC using genome-wide microarray data and Single-cell RNAseq data from hippocampal Nestin-GFP NSCs (Shin et al., 2015). The *X* axis refers to the number of upregulated genes in each pathway. The Lef1 annotation indicates that *Lef1* is present among the Wnt pathway genes upregulated in quiescent NSPCs. **(B)** Heatmap illustrating the expression of Wnt signalling pathway genes obtained from the microarray transcriptomic data in A-NSPC and Q-NSPC. Blue and red color intensities indicate down and upregulated genes, respectively. Columns represent samples from independent experiments. **(C)** Quantitative RT-PCR validation in independent samples of Wnt pathway genes selected from the microarray transcriptomic data (Lef1 (*n* = 8, *p* < 0.01), Fzd8 (n = 5, *p* < 0.05), Gsk3b (*n* = 4, *p* < 0.01, *t*-test). **(D)** *Upper panel*, Schematic description of the lentiviral 7xTCF/LEF1-eGFP/SV40-mCherry reporter. *Lower panel*, fold increase in eGFP/mCherry relative RNA levels in transfected A- and Q-NSPCs as determined by RT-qPCR (*n* = 4, Mann Whitney *U*-Test, *p* < 0.05. **(E)** Active β-catenin protein level analyzed by Western blot in A- and Q-NSPCs. β-actin was used as loading control (2.9-fold increase, *n* = 4, *t*-test, *p* < 0.05. **(F)** Confocal images showing the Tuj1+ neurons generated after 4DIV of treatment of A- and Q-NSPCs with 1.5 μM CHIR99021 (abbreviated as CHIR) compared to control conditions. **(G)** Quantification of the percentage of neurons (Tuj1+) generated in A- and Q-NSPCs cultures upon 4 days *in vitro* (4DIV) differentiation at increasing CHIR doses (*n* ≥ 4, *p*-values determined by one-way ANOVA referred to control untreated cells A-NSPCs *p* < 0.01, Q-NSPCs *p* < 0.01) or two-way ANOVA (cell type *p* < 0.0001 and treatment *p* < 0.001). The %Tuj1+ cells in A- and Q-NSPCs cultures prior to the differentiation protocol (0DIV) is shown as a reference. **(H)** *Lef1* and *Id1* mRNA induction kinetics as analyzed by RT-qPCR upon exposure to FGF2+BMP4 (quiescence induction; Mean ± SEM values from A-NSPC (*n* = 3) Q-NSPC (*n* = 4) and five independent kinetics, one-way ANOVA (Lef1 48, 72, 96 h *p*-values 0.01, <0.01, <0.05 respectively; Id1 12 h *p* < 0.01). **(I)** LEF1 protein levels detected by Western blot in A- and Q-NSPCs (extracts from three independent experiments are shown). Two LEF1 bands were observed. **(J)** Quantification of the relative accumulation of total LEF1 (*n* = 7, *t*-test *p* < 0.05, LEF1 upper band (*n* = 4, *t*-test *p* < 0.01) and LEF1 lower band in Q-NSPCs vs. A-NSPCs. β-actin was used as loading control. Data are presented as Mean ± SEM, except in D, where data are presented as Median ± interquartile range. *p < 0.05; **p < 0.01; ***p < 0.001; ****p < 0.0001 Scale in F: 25 μm.

To further obtain biological insight into the transcriptomic data, we next focused on the signalling pathway categories provided by KEGG. The analysis revealed an enrichment in MAPK, Wnt, Neurotrophin, PPAR and Insulin pathways in quiescent NSPCs that was shared by quiescent adult hippocampal NSCs *in vivo*, based on a previously published single-cell RNAseq analysis (Shin et al., 2015) (Figure 2A). Each pathway entity contained key genes encoding receptors, ligands and/or downstream signalling mediators. Of note, many of the aforementioned pathways play relevant roles during adult neurogenesis. This has led to propose that quiescent stem cells in the hippocampal dentate gyrus are primed or predisposed to respond to changes in their local microenvironment, possibly displaying an enhanced niche signal integration capacity, and that, once activated, the stem cells shunt their capacity to respond to external regulation (Shin et al., 2015). To explore putative differences in signal transduction between quiescent and active NSPCs, we next focused on the Wnt signalling pathway, a paradigmatic pathway in the regulation of adult hippocampal neurogenesis that specifies the neuronal fate of the hippocampal stem cell progeny (Lie et al., 2005; Kuwabara et al., 2009; Qu et al., 2013; Heppt et al., 2020; Austin et al., 2021).

### Enhanced Wnt signalling and *Lef1* induction in quiescent adult hippocampal NSPCs

Several components of the canonical Wnt signalling pathway were significantly induced in the quiescent NSPCs, including ligands (*Wnt10a*), receptors (*Fzd8*), downstream mediators of the canonical pathway (*Gsk3b, Apc*) and most importantly, the transcriptional activator *Lef1* and the bona fide TCF/LEF1 target gene *Axin2,* suggesting that Q-NSPCs are endowed with a basal induction of canonical Wnt signalling (Figure 2B, microarray transcriptomic data; Figure 2C; validation in independent samples by RT-qPCR). Indeed, when Q- and A-NSPCs were transduced with a lentiviral vector carrying the 7xTCF/LEF1-eGFP, a reporter for TCF/LEF1 activity, followed by an SV40-mCherry reference cassette (Fuerer and Nusse, 2010), we found that TCF/LEF1-driven transcription was significantly enhanced in the quiescent state compared to actively proliferating cells (>2-fold increase, Figure 2D, *p* < 0.05; Supplementary Figure S1G). We next assessed if this moderate raise in baseline TCF/LEF1 activity correlated with an increase in non-phospho (active) β-catenin, employing an antibody designed to specifically recognize the stabilized form of β-catenin (not phosphorylated at the conserved Ser^33^, Ser^37^, and Thr^41^ residues by GSK3B) that is functionally active in the canonical Wnt pathway. Accordingly, we found that active β-catenin accumulated in Q-NSPCs compared to A-NSPCs (Figure 2E and Supplementary Figure S2, *p* < 0.05).

We then explored whether this basal TCF/LEF1 activity and active β-catenin accumulation rendered the quiescent NSPCs more responsive to the canonical Wnt pathway. To this end, we employed a previously reported assay that is sensitive to Wnt signalling (Lie et al., 2005; Michaelidis and Lie, 2008; Varela-Nallar and Inestrosa, 2013; Choe et al., 2015; Armenteros et al., 2018). We exposed Q- and A-NSPCs to increasing doses of the GSK3B inhibitor CHIR99021, that functions as a canonical Wnt activator, and we differentiated the cells for 4 DIVs in the absence of mitogenic stimulation to follow neuronal production as a readout of enhanced Wnt signalling (Figures 2F,G). Immunocytochemistry analysis showed that, upon differentiation, Q-NSPCs generated significantly more TuJ1^+^ cells than A-NSPCs in culture even in the absence of CHIR99021. Both Q- and A-NSPCs responded in a dose-dependent manner to CHIR99021 stimulation, producing significantly more TuJ1^+^ cells than untreated NSPCs (Figure 2G). In accordance to this, active β-catenin accumulated in A- and Q-NSPCs upon CHIR99021 exposure (Supplementary Figure S3A,B). Statistical analysis by 2-way ANOVA revealed that both the factor “treatment” (CHIR) and the factor “cellular state” (Q/A) had a significant effect (F_2,23_ = 13.25, *p* < 0.001 and F_1,23_ = 59.78, *p* < 0.0001, respectively) although the “treatment-state” interaction was not significant (*p* = 0.2772). Thus, the data imply that Q-NSPCs have an increased Wnt signalling activity in basal conditions and upon stimulation of the pathway employing the GSK3B inhibitor CHIR99021. Of note, the increased baseline generation of TuJ1+ cells by the Q-NSPCs was surprising since *in vivo*, in the adult hippocampal neurogenic lineage, quiescent NSCs need to become active and express proneural genes such as *Ascl1* prior to their differentiation. Thus, the enhanced neuronal production by Q-NSPCs when cultures are switched to differentiation conditions may be related to the *in vitro* set-up. Further investigation is required to clarify whether and how neuronal commitment of cultured Q-NSPCs is increased at a mechanistic level.

We next turned our attention to the transcription factor LEF1, given its pivotal role in the transactivation of Wnt/β-catenin responsive genes, its prominent function in the development of the dentate gyrus (Galceran et al., 2000; Zhou et al., 2004) and its overexpression at the mRNA level in quiescent adult hippocampal NSCs *in vitro* and *in vivo* (Figure 2A). We first examined the *Lef1* induction kinetics during the entry of the NSPCs into quiescence by RT-qPCR (Figure 2H). Compared to the BMP target gene *Id1*, that was transiently induced very early after the exposure of the cells to BMP4, *Lef1* gene expression progressively increased coinciding in time with the entry of the NSPCs into the G_0_ quiescent state in our cell culture system. We then confirmed by Western blot that the upregulation of *Lef1* transcription was paralleled by the accumulation of LEF1 at the protein level (Figures 2I,J). Two LEF1 protein bands that differed in about 4 kDa were detected (Figures 2I,J and Supplementary Figure S3C and 4). Accumulation of the upper one accounted for the increase in total LEF1 protein in Q-NSPCs (Figures 2I,J) and seemed unrelated to protein modifications by phosphorylation (Supplementary Figure S3E,F). These results pointed to an additional layer of regulation in *Lef1* expression through differential post-transcriptional events.

### *Lef1* alternative splicing and enhanced LEF1 stability in quiescent adult hippocampal NSPCs

**FIGURE 3:**
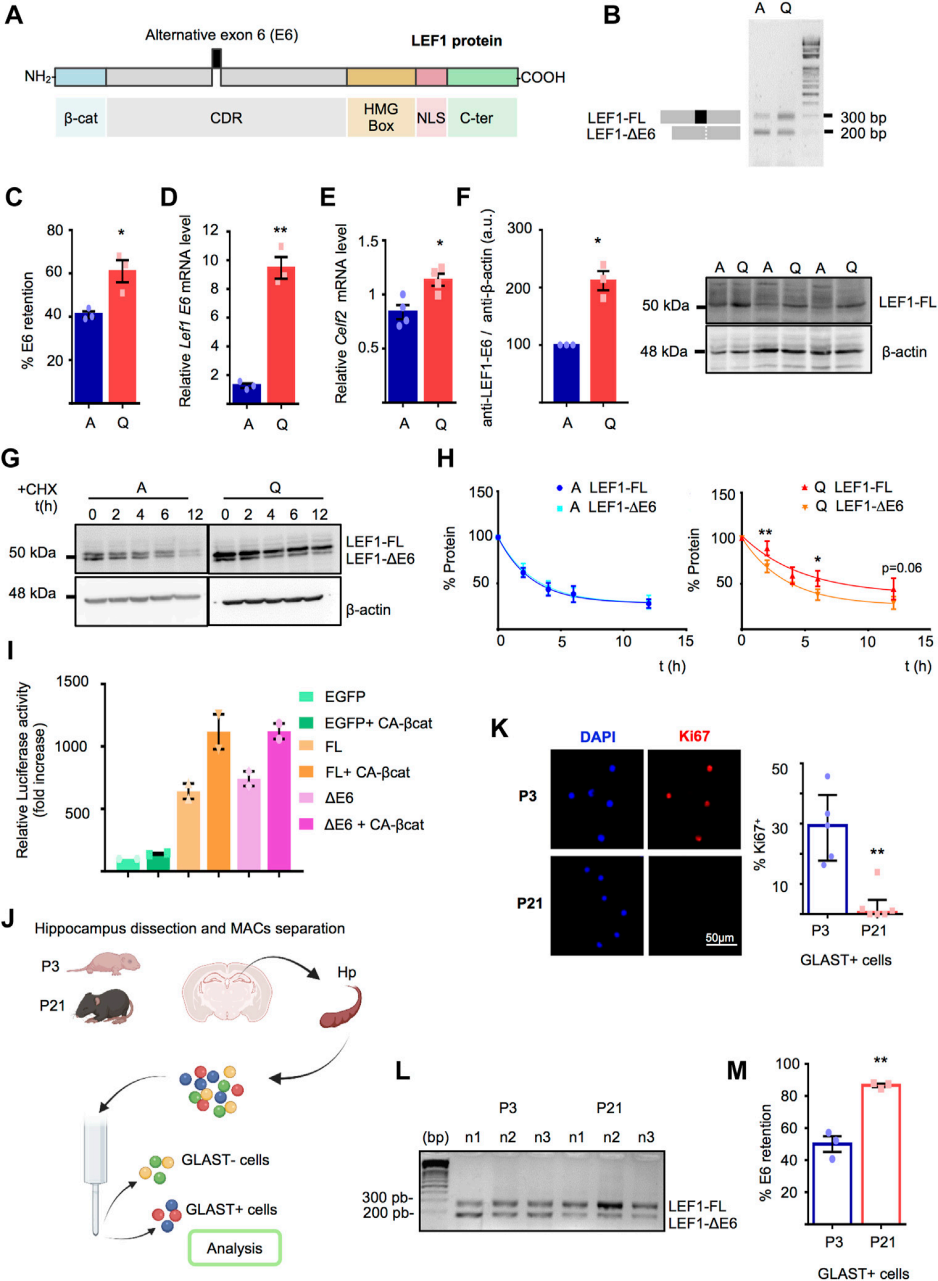
(A) LEF1 protein domains showing the location of the residues affected by the E6 alternative splicing within the context-dependent regulatory domain (CRD). (B) Representative example of the Lef1 RT-PCR employing primer pairs flanking E6 and cDNA from A- and Q-NSPCs. The PCR products corresponding to Lef1-FL and Lef1-ΔE6 were resolved by agarose gel electrophoresis. (C) Percentage of Lef1 E6 retention in Q-NSPCs vs. A-NSPCs (n = 3, t-test, p < 0.05) (D) Validation of Lef1 mRNA expression by RT-qPCR employing E6 internal primer sets (>8-fold increase in Q-NSPCs vs. A-NSPCs, t-test, p < 0.01). (E) Relative Celf2 expression in Q-NSPCs vs. A-NSPCs by RT-qPCR (n = 4, t-test, p < 0.05. (F) Right, Western Blot of A- and Q-NSPCs showing LEF1-FL by using an antibody raised against a conserved epitope mapping the CRD residues encoded by E6. Left, quantification of LEF1-FL levels normalized to β-actin (n = 3, one sample t-test, p < 0.05). (G) LEF1 protein stability assays in A- and Q-NSPCs. Cells were treated with cycloheximide (CHX) and the stability of the upper and lower band was measured by Western blot. β-actin was used as loading control. (H) Percentage of LEF1-FL or LEF1-ΔE6 in the protein stability assays performed in A- and Q-NSPCs. Note the enhanced stability of LEF1-FL in quiescence (n = 4). t (h), hours upon CHX addition. (I) Relative 7xTCF reporter luciferase activity (fold increase) in Neuro2A cells overexpressing EGFP, LEF1-FL or LEF1-ΔE6 with or without a aconstitutive active (CA) β-catenin (n = 2). (J) Diagram illustrating the isolation of GLAST+ cells from the hippocampus of wild type mice at postnatal day 3 (P3) and 21 (P21). (K) Left panel, representative confocal images showing the Ki67+ GLAST+ cells from postnatal P3 and P21 oldmice. Right panel, quantification of the percentage of Ki67 in P3 and P21 GLAST+ cells (n ≥ 5, Mann Whitney U-Test, p < 0.01). (L) Agarose gel electrophoresis showing the RT-PCR products of Lef1-FL and Lef1-ΔE6 from P3 and P21 GLAST+ cells. (M) Percentage of Lef1 E6 retention in P3 and P21 GLAST+ cells (n = 3, t-test, p < 0.01). Data are presented as Mean ± SEM, except in K, where data are presented as Median ± interquartile range. *p < 0.05; **p < 0.01; ***p < 0.001. Diagram in (I) was created with BioRender.com.

LEF1 is a multidomain protein composed of a N-terminal domain that binds to β-catenin, a context-dependent regulatory domain (CRD) that interacts with both transcriptional repressors and activators, a High Mobility Group (HMG) DNA-binding domain that recognizes the Wnt Response Element motif (WRE: 5′-CTTTGWW-3′), a nuclear localisation signal and a C-terminal domain (Figure 3A). Splicing events in the *Lef1* gene leading to rearrangements in LEF1 protein domains have been previously reported (Arce et al., 2006; Mallory et al., 2011; Nagalski et al., 2013). Given “spliceosome” was among the gene categories enriched in A-NSPCs, we set out to investigate whether alternative splicing could account for the differences in the LEF1 protein profile observed by Western blot. *Lef1* mRNA splicing in Q- and A-NSPCs was initially explored through RT-PCR analysis of all the *Lef1* exons employing primer pairs flanking each exon. This revealed a skipping of E6 affecting a portion of the CRD in NSPC cultures (Figure 3B and Supplementary Figure S5). The identity of the spliced amplicon was corroborated through purification and sequencing (Supplementary Figure 5A,B). No alternative splicing events were observed for other *Lef1* exons (Supplementary Figure S5C). We found that *Lef1* mRNA in Q-NSPCs showed a significant increase in E6 inclusion (% E6 retention: 61 ± 5%) compared to A-NSPCs (% E6 retention: 41 ± 1%; *p* < 0.05, Figure 3C) that was further confirmed by RT-qPCR employing E6 internal primer sets (8-fold increase; *p* < 0.01, Figure 3D). Together, this indicates that Q-NSPCs are enriched in a *Lef1-Full Length* mRNA isoform (*Lef1-FL*) compared to A-NSPCs. Variable inclusion of *Lef1* E6 has been previously studied in thymocytes. In a human T-cell line, it has been reported that E6 inclusion in response to activation signals is required for the optimal induction of LEF1 target genes and that E6 retention depends on the expression and binding of CELF2 protein to sequences flanking E6 (Arce et al., 2006; Mallory et al., 2011; Nagalski et al., 2013). Therefore, we evaluated *Celf2* expression in the NSPC cultures by RT-qPCR (Figure 3E). We found a significant raise *Celf2* transcript abundance in Q-NSPCs.

We next corroborated the accumulation of LEF1-FL in Q-NSPCs at the protein level using an antibody raised against a conserved epitope mapping the CRD residues encoded by E6 (Figure 3F and Supplementary Figure S5D). We also compared the stability of the two LEF1 protein isoforms in quiescent and actively dividing NSPCs. Half-life (t_1/2_) assays employing cycloheximide to block translation demonstrated an enhanced stability of the LEF1 larger form in the quiescent state, favouring its accumulation, while both isoforms showed similar stability in the active state (Figures 3G,H, A-NSPCs: LEF1-FL t_1/2_ = 1.9 h and LEF1-ΔE6 t_1/2_ = 2.0 h; Q-NSPCs: LEF1-FL t_1/2_ = 3.3 h and LEF1-ΔE6 t_1/2_ = 2.4 h; *p* < 0.01; see also Supplementary Figure S6). Finally, we compared the transactivation capacity of the two LEF1 protein isoforms employing the 7xTCF-luciferase Optimal Promoter (TOPflash) reporter assays. When LEF1-FL or LEF1-ΔE6 were overexpressed in Neuro2A together with a constitutive active form of its Wnt signalling partner β-catenin (CA-β-cat), both isoforms acted as transcriptional activators and performed equally in terms of eliciting the canonical Wnt signalling pathway (Figure 3I).

Next, we explored if E6 retention was also regulated *in vivo* at the time hippocampal NSCs enter the quiescent sate. Recently, it has been demonstrated that during postnatal development, proliferation of NSCs in the hippocampal dentate gyrus peaks at postnatal (P) day P3. This is followed by a transition of the cells into a radial glia-like quiescent state occurring around the second postnatal week (Berg et al., 2019). Astroglia and hippocampal radial glia-like neural stem cells express the glutamate/aspartate transporter (GLAST) (DeCarolis et al., 2013). Thus, we prospectively isolated GLAST^+^ cells from the hippocampus of P3 and P21 days-old mice (Figure 3J). Immunocytochemistry analysis of the isolated cells with the cell cycle marker Ki67 confirmed that hippocampal GLAST^+^ cells were cycling at P3 and abandoned the cell cycle at P21 (Figure 3K, *p* < 0.01). We then investigated the alternative splicing of *Lef1* E6 in the P3 and P21 GLAST^+^ cell populations by RT-PCR (Figure 3L). Two *Lef1* mRNA isoforms (*Lef1-FL* and *Lef1-ΔE6*) were detected in the isolated cells, similar to the NSPC cultures. Besides, GLAST^+^ P21 cells exhibited a significant increase in E6 inclusion (% E6 retention: 86 ± 1%) relative to GLAST^+^ P3 cells (% E6 retention: 50 ± 5%; *p* < 0.01, Figure 3M), suggesting a preponderance of the *Lef1-FL* mRNA isoform upon the entry of postnatal GLAST^+^ hippocampal cells into the quiescent state *in vivo*. Since the transition of the proliferative radial glia-like GLAST^+^ cells to quiescence takes place concomitantly with astrogliogenesis in the postnatal hippocampus, we cannot exclude that the change in isoform may reflect a change in the number or maturation of the GLAST^+^ astrocytes at P21 vs. P3.

In summary, the alternative E6 splicing in NSPCs gives rise to two mRNA species, differing in 84 nucleotides, and is markedly regulated in the transition from proliferation to quiescence. The mRNA including E6 preferentially accumulates in quiescent NSPCs and encodes a full-length protein isoform (LEF1-FL) that is more stable. The mRNA lacking E6 codes for a shorter LEF1 isoform lacking part of the CRD domain codified by E6 (28 aa; LEF1-ΔE6) and is expressed similarly in both actively proliferating and quiescent NSPCs. Both isoforms activate canonical Wnt signaling reporters.

### LEF1 binding motifs enriched in NSPC enhancers and putative LEF1 targets in quiescent NSPCs

**FIGURE 4:**
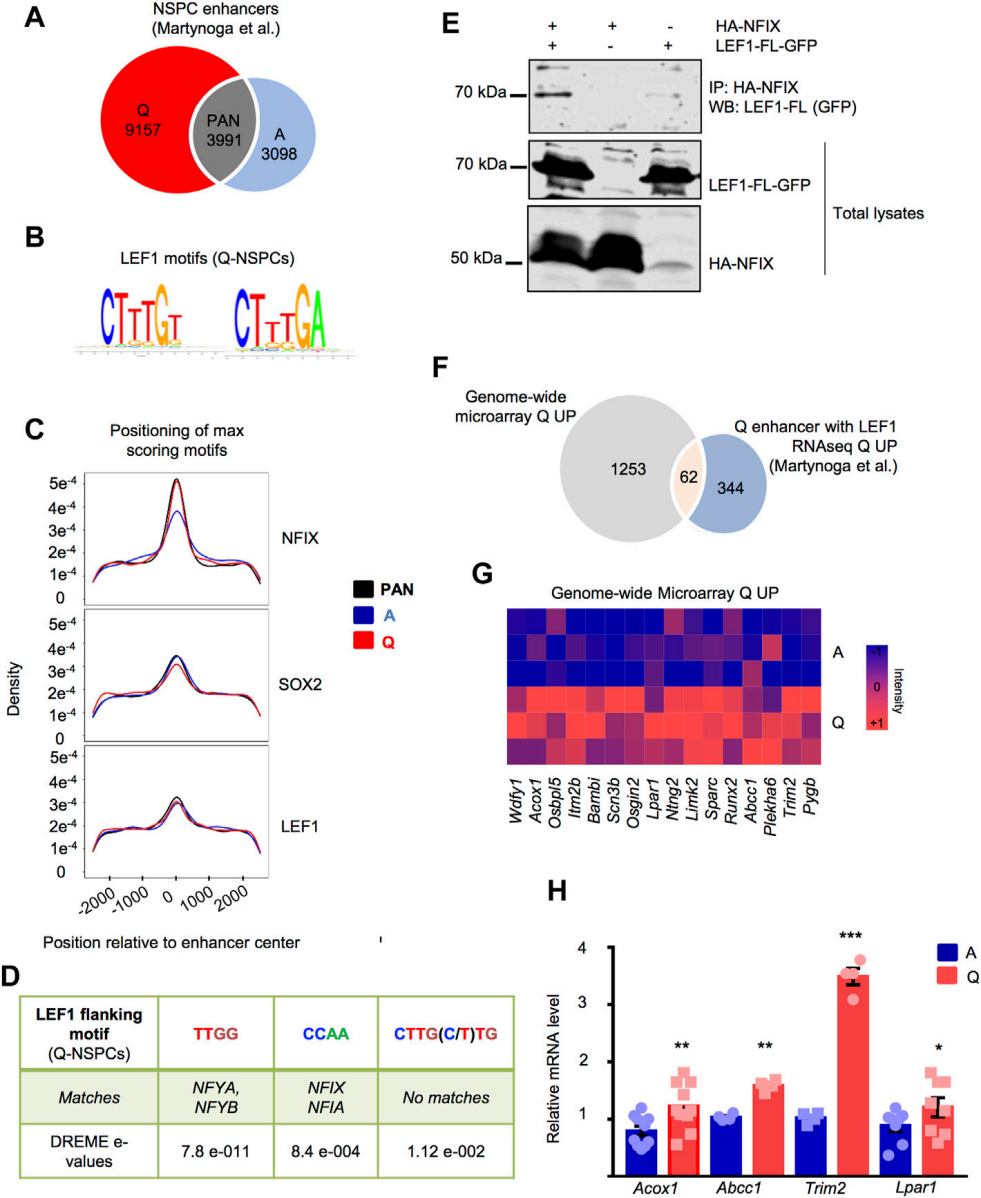
**(A)** Venn diagram showing the distribution of the NSPC enhancers identified in quiescent (Q), active (A) and both (PAN) NSPCs by ChIP-seq in Martynoga et al. (2013) that were analyzed for LEF1 motif enrichment. **(B)** Position weight matrix for the LEF1 motifs enriched in NSPC enhancers. **(C)** Enrichment of the NFIX, SOX2 and LEF1 motifs in A-NSPC, Q-NSPC and PAN enhancer groups. **(D)** Transcription factor binding motifs significantly enriched in sequences flanking the LEF1 motifs of Q-NSPC enhancers, as analyzed by DREME *de novo* motif discovery. **(E)** Co-immunoprecipitation (Co-IP) and Western blot analysis of the HA-tagged NFIX and GFP-tagged LEF1-FL expressed in HEK293 cells, showing the NFIX/LEF1 interaction. A representative experiment is shown. The Co-IP was repeated in *n* = 7 independent experiments. **(F)** Venn diagram showing the number of upregulated genes in Q-NSPCs with LEF1 motifs in their closest enhancer according to the Martynoga et al. (2013) RNAseq data and the genome-wide microarray transcriptomic data from the current study. **(G)** Heatmap illustrating the overexpression in Q-NSPCs of the 16 genes that showed LEF1 motifs in their closest enhancer exclusively in the quiescent state. **(H)** Validation in independent samples of a selection of genes from the list in G by RT-qPCR (fold increase in Q-NSPCs vs. A-NSPCs) for *Lpar1* (*n* = 8, *p* < 0.05), *Acox1* (*n* = 10, *p* < 0.05), *Abcc1* (*n* = 4, *p* < 0.01), *Trim2* (*n* = 4, *p* < 0.01) determined by *t*-test. Data are presented as Mean ± SEM. *p < 0.05; **p < 0.01; ***p < 0.001.

We set out to explore the enrichment in LEF1 binding motifs in the enhancers of the NSPCs. We took advantage of a previously generated dataset that located the histone acetlytransferase p300 and the histone modification H3K27ac in the NSPC genome through chromatin immunoprecipitation coupled to DNA sequencing (ChIP-seq data from Martynoga et al., 2013). In this dataset, active enhancers were identified in BMP4-induced quiescent and actively proliferating NSPCs (NS5 cells (Conti et al., 2005)). Among them, 9,157 were quiescence-specific (Q), 3,098 were active-specific (A) and 3,991 were pan-NSPC enhancers that were equally present in both conditions (PAN) (Figure 4A).

We searched this enhancer dataset for LEF1 motifs retrieved from public collections and analysed their enrichment in the NSPC enhancer regions versus flanking sequences, as well as between the different Q, A and PAN enhancer groups. We found that the 5′-CTTTGT-3′ LEF1 motif, and to a lesser extent the 5′-CTTTGA-3′ motif, was over-represented in enhancer vs. flanking sequences in the three NSPC enhancer groups (bootstrap *p*-value < 0.05). However, there was no clear evidence for a preferential enrichment of the LEF1 motif in any of the Q, A and PAN categories (Figures 4B,C). No apparent bias at any motif position was found in the Q, A and PAN enhancers. Similarly, the motif bound by SOX2 [5′-CC(TA)TTGT(TGC)-3′], a transcription factor that primes the epigenetic landscape in neural precursors to enable adequate gene activation during hippocampal neurogenesis (Amador-Arjona et al., 2015), was enriched in all the NSPC enhancers although it was mildly over-represented in the A and PAN enhancer groups (Figure 4C). In line with the previous enhancer analysis (Martynoga et al., 2013), some other motifs were over-represented in particular enhancer groups compared to other (bootstrap *p*-value < 0.05). Specifically, NFIX motif (consensus sequence 5′-TTGGCA-3′) was enriched in quiescent-specific enhancers compared to active-specific enhancers (Figure 4C), whereas both ASCL1 (5′-GCAGCTG-3′) and ASCL2 (5′-CAGCTGC-3′) motifs showed the opposite pattern and were enriched preferentially in A enhancers (Supplementary Figure S7).

We further explored all three categories of NSPC enhancers employing DREME *de novo* motif discovery (Bailey, 2011) to search the flanking sequences of the 5′-CTTTGT-3′ LEF1 motif (15bp, (+) strand always). DREME output was used as input to the TOMTOM tool (Gupta et al., 2007). We found significant enrichment for predicted binding sites for the transcription factors NFYA, NFYB, NFIA, and NFIX in the LEF1 flanking sequences of the Q versus A enhancers and in both of them versus background sequences (*p* < 0.01) (Figure 4D). In summary, the genomic regions with epigenetic characteristics of active enhancers in both quiescent and actively dividing NSPCs are enriched in LEF1 transcription factor DNA-binding motifs. In enhancers that are specifically active in the quiescent state, the LEF1 flanking sequences are enriched in putative NFY and NFI motifs. Thus, LEF1 could be potentially interacting with NFY and NFI transcription factors in the quiescence-specific enhancers to regulate nearby genes. Indeed, when transfected into HEK293T cells, NFIX-HA and LEF1-FL-GFP co-immunoprecipitated, indicating that NFIX and LEF1 can establish protein-protein interactions (Figure 4E and Supplementary Figure S8). NFIX-HA was also able to co-immunoprecipitate with LEF1-ΔE6 (Supplementary Figure S7B,C), suggesting that the interaction does not necessarily require the CRD region encoded by E6.

We next evaluated the relationship between the changes in gene expression and the presence of the TCF/LEF1 motif in the closest enhancer. Using the RNA-seq and ChIP-seq data from the Martynoga et al. study, we tested whether genes with at least one enhancer that has a LEF1 motif are more likely to be differentially regulated. Out of the genes putatively regulated by quiescence enhancers that were overexpressed in quiescence in the Martynoga et al*.* study, 62.5% had a LEF1 motif (Jaspar ID PF0013.1; 406 genes, Supplementary Table S3). This enrichment, although modest, was significant (*p* = 8 × 10^–5^, Chi-squared test). The same comparison with genes that were putatively regulated by A-specific or PAN enhancers was not significant (*p* > 0.05, Chi-squared test). Among these 406 upregulated genes, we encountered genes expressed by hippocampal dentate gyrus precursors throughout embryonic and postnatal development (*Hopx*) (Berg et al., 2019), as well as genes enriched in quiescent adult radial-glia like NSCs (*Id4*) (Blomfield et al., 2019; Zhang et al., 2019). Out of the 406 genes, 126 showed the LEF1 motif *exclusively* in the Q enhancers (this is, they lacked the motif in the PAN and A enhancers, Supplementary Table S3; note that 115 out of the 126 genes had both LEF1 and NFIX motifs at one or more Q-specific enhancers). Functional enrichment with Gene ontology (GO) of the 126 genes is provided in Supplementary Figure S9. Moreover, out of the 406 genes, 62 were significantly overexpressed as well in our study (Figure 4F, Supplementary Table S3). We focused on 16 of these common upregulated genes that showed a LEF1 motif *exclusively* in the quiescence enhancer list (Figure 4G, Supplementary Table S3). We further validated the upregulation of a selection of these genes by RT-qPCR in independent samples (Figure 4H), including *Lpar1*, an important cell surface marker expressed by hippocampal NSPCs *in vivo* that allows their prospective isolation (Walker et al., 2016). We confirmed that *Lpar1* gene had both LEF1 and NFIX motifs in its putative Q-specific enhancer (Martynoga et al., 2013).

## Discussion

To better understand adult neural stem cell behaviour at a more refined molecular level, we have investigated the transcriptome of quiescent versus active adult hippocampal neural NSPCs. Notably, the gene expression profile of quiescent NSPCs in culture closely resembled that of their *in vivo* counterparts, as reported by single-cell RNAseq of Nestin-GFP animals (Shin et al., 2015). Previous transcriptomic studies of quiescent NSPCs dealt with metabolic, cell adhesion and proteostatic changes (Llorens-Bobadilla et al., 2015; Shin et al., 2015; Leeman et al., 2018; Morizur et al., 2018). Instead, our study focused on the enrichment in signalling pathway components and aimed to provide functional evidence for the putative priming of the adult quiescent stem cells. It has been previously proposed that quiescent radial glia-like NSCs in the hippocampal dentate gyrus are better equipped to respond to changes in their local microenvironment. The Shin *et al.* dataset, for instance, revealed high expression of several signalling pathway-related genes in quiescent hippocampal NSCs and their downregulation during the initial stages of adult hippocampal neurogenesis, including the Wnt pathway and the *Lef1* gene (Shin et al., 2015). LEF1 lies at the core of the canonical Wnt pathway and is considered as a landmark transcription factor expressed by the hippocampal DG NSCs throughout development and during adult stages (Choe et al., 2013). Thus, we chose to focus on Wnt and LEF1, and we designed a series of experiments to explore whether quiescent NSPCs *in vitro* are predisposed respond to this signalling pathway.

We herein demonstrate that adult hippocampal quiescent NSPCs cultured *in vitro* overexpress Wnt related genes compared to proliferating NSPCs. In our experimental set-up, we employed FGF2 to expand rat NSPC neurosphere cultures and treated them with BMP4 to induce quiescence. In other studies, adherent adult hippocampal mouse NSPC cultures supplemented with both EGF and FGF2 have been employed to support NSPC proliferation and BMP4 has been used to promote quiescence. Wnt related genes are also overexpressed in the latter murine set-up; for instance, in the study by Austin et al. (2021), using the published bulk RNA-sequencing dataset from Blomfield et al. (2019) it has been reported that quiescent adherent mouse NSPCs upregulate the expression of Wnt receptors, transducer molecules including β-catenin and Wnt ligands. This gene expression profile largely recapitulates both the *in vitro* results of the current study and the expression of Wnt pathway components observed in quiescent and active NSCs in mice *in vivo* (Shin et al., 2015; see Figure 2A). In addition, in the current study we most importantly show that quiescent NSPCs are endowed with a basal induction of canonical Wnt signalling. This was measured employing TCF/LEF1 activity reporter assays and was corroborated through the accumulation of the active (non-phosphorylated) form of β-catenin. We provide further insight by showing that despite the increase in basal Wnt signalling, Q-NSPCs do not display a greater response to increasing GSK3B inhibition compared to A-NSPCs. The accumulation of activated β-catenin over time seems to plateau at the same level upon GSK3B inhibition, although the rise seems perhaps slightly faster in quiescent cells. Our data thus indicate that the Wnt signalling machinery is working similarly in Q- and A-NSPCs, although starting from a higher baseline in the quiescent state. We thus partly corroborate recent findings showing state-specific responses of quiescent and active neural stem cells to Wnt/β-catenin signalling (Austin et al., 2021). Since the Wnt signalling pathway cooperates with many other pathways, including Notch and BMP (Itasaki and Hoppler, 2010; Muñoz Descalzo and Martinez Arias, 2012; Armenteros et al., 2018), basal differences in Wnt activity may in turn impact in the way quiescent and active stem cells integrate other cues emanating from the niche.

Interestingly, at a mechanistic level, our results differ from a study addressing the regulation of Wnt signalling in other quiescent adult stem cell populations such as muscle stem cells (MuSCs) (Aloysius et al., 2018). It has been reported that TCF/LEF1-mediated transcription is upregulated in quiescent myoblasts and supressed upon their reactivation (Subramaniam et al., 2014); however, the TCF/LEF1 signalling in the quiescent myoblasts is independent of β-catenin and instead relies on the transcriptional co-activator SMAD3, a downstream effector of the TGFβ pathway. This indicates that in quiescent myoblasts LEF1 swaps partners from β-catenin to SMAD3 in order to regulate TCF/LEF1 target genes, a phenomenon that also takes place *in vivo* when postnatal MuSCs transition from proliferation to quiescence. In contrast, we have found that the levels of active non-phosphorylated β-catenin are increased in quiescent NSPCs (while they are reduced in quiescent myoblasts/MuSCs). Thus, contrary to quiescent stem cells in the muscle, the TCF/LEF1 signalling that characterises the quiescent state of adult stem cells in the neural lineage seems to be Wnt- and β-catenin dependent, at least *in vitro*.

The activity of canonical Wnt signalling in the adult hippocampal NSC niche has been previously analysed *in vivo* taking advantage of several mouse reporter lines. In BATGAL mice with the LacZ reporter gene downstream of 7xTCF/LEF1 binding sites (Maretto et al., 2003) approximately 25% of the radial glia-like adult NSCs were LacZ^+^ (Heppt et al., 2020). A fraction of the intermediate progenitor cells was also LacZ^+^ although cells had lower reporter levels than NSCs. This trend was corroborated in Axin2^LacZ/+^ mice that harbor the LacZ reporter in the endogenous *Axin2* locus, a reliable Wnt signalling target gene (Lustig et al., 2002; Heppt et al., 2020). Given LEF1-expressing cells in the adult DG are mostly radial glia-like NSCs (Choe et al., 2013), and since this population is primarily (>95%) out of the cell cycle during adulthood, probably LEF1 protein accumulates *in vivo* in the quiescent NSCs of the adult hippocampal niche. The presence of LEF1 in quiescent NSCs is also supported by the increase in *Lef1* expression at the mRNA level detected in quiescent NSCs isolated from the hippocampi of Nestin-GFP animals, as analysed by single-cell RNAseq (Shin et al., 2015). Nevertheless, a recent study showed no correlation between LacZ levels in NSCs from BATGAL mice and activation/quiescence markers, indicating that NSCs *in vivo* respond to Wnt regardless of their cellular state (Austin et al., 2021).

The higher basal activity of canonical Wnt signalling in quiescent NSPCs *in vitro* may be due at least in part to the combined upregulation of *Lef1* transcription and E6 inclusion by alternative splicing, that lead to the accumulation of a full-length LEF1 protein isoform endowed with increased stability in quiescence. Taken together, these events imply that quiescent NSPCs are equipped with higher levels of a more stable LEF1 protein. Of note, both the LEF1-FL and LEF1-ΔE6 isoforms behaved as transcriptional activators in cooperation with β-catenin. Functional diversity of the TCF/LEF1 family of transcription factors is partly achieved by alternative splicing (Arce et al., 2006). Indeed, E6 alternative splicing is a conserved event in many species [see Supplementary Table S5 including information from the VastDB database of Alternative Splicing (Tapial et al., 2017)]. Interestingly, all vertebrate members of the TCF/LEF1 family have an alternative exon in the context dependent regulatory domain (CRD) region. The CRD has been mainly involved in transcriptional repression by recruiting the pleiotropic repressor Groucho (transducin-like enhancer of split factor, TLE, in human) although a role for CRD in cooperative interactions with transcriptional activators has been also proposed (Arce et al., 2006). The exact function of the CRD/E6 in LEF1 is still largely unknown. Some examples show that expression of the alternatively spliced *Lef1* isoforms can dictate a switch in cellular behaviour. During thymus development, for instance, an increase in E6 inclusion contributes to the activation of T cell antigen receptor α enhancer, the most critical checkpoint in T cell maturation (Mallory et al., 2011). In pancreatic carcinoma cells, transient overexpression of the FL isoform induced cell cycle-related genes (encoding c-myc and cyclin D1) in cooperation with β-catenin. Instead, overexpression of LEF1-ΔE6 inhibited E-cadherin expression independent of β-catenin and enhanced cell migration (Jesse et al., 2010). In contrast, other reports indicate that the shorter LEF1 isoform lacking E6 is devoid of the binding site for HIC5, a repressor of β-catenin-dependent function (Ghogomu et al., 2006).

E6 skipping affecting the CRD of the *Lef1* gene has been also reported in the nervous system (Nagalski et al., 2013). In the adult brain, *Lef1* expression has been detected in NSC niches and in postmitotic thalamic and dorsal midbrain neurons. In the thalamus, *Lef1* E6 exhibited a strong developmental regulation, with E6 inclusion increasing from embryonic day E18.5 to P60. The functional implication of this developmental switch is still unknown. We now provide evidence for a similar alternative E6 splicing regulation in GLAST^+^ cells isolated form the P3 to P21 postnatal hippocampus. Furthermore, in adult hippocampal NSPC cultures we found that, compared to the truncated LEF1 isoform lacking the residues encoded by E6, the LEF1-FL isoform that preserves an intact CRD has a higher intrinsic stability in quiescence. This suggests that this protein region protects LEF1-FL from degradation through a yet unknown mechanism. Interestingly enough, the stretch of residues encoded by E6 includes the conserved Ser^229^, Ser^230^, Ser^232^, Ser^236^, and Ser^238^ that are potential SSXS and SXS phosphorylation sites. LEF1 is phosphorylated upon Wnt signalling activation by the HIPK2 kinase in the HMG box domain (Hikasa et al., 2010; Hikasa and Sokol, 2011) and in the central region by nemo-like kinase (Ishitani et al., 2003), a modification that triggers its ubiquitination and proteasomal degradation (Yamada et al., 2006). Given the increased stability of LEF1-FL in Q-NSPCs, it is tempting to speculate that phosphorylation of E6-encoded Ser residues would instead protect LEF1 from its degradation. LEF1-FL in quiescence perhaps interacts with additional factors that preclude the ubiquitination and degradation of the protein. Future studies should address the molecular basis for the increase in LEF1-FL stability in Q-NSPCs.

Locating where transcription factors such as LEF1 bind to the genome is the key to understand gene regulation. We initially hypothesized that if LEF1 plays a relevant role in quiescent cells, quiescence-specific enhancers should be enriched in LEF1 DNA-binding motifs. Nevertheless, we did not find such differential enrichment; we found instead active enhancers with LEF1 motifs in Q, A and PAN enhancers. Nevertheless, when we focused only on the genes putatively regulated by quiescence enhancers that were overexpressed in quiescence in the Martynoga et al. study, we did find an enrichment as 62.5% of them had a LEF1 motif. Similarly, the motif bound by the NSC transcription factor SOX2 [5′-CC(TA)TTGT(TGC)-3′] was enriched in all enhancer categories, although it was mildly over-represented in the A and PAN enhancer groups. LEF1 and SOX2 are representatives of a class of HMG domain proteins that recognize variants of the consensus nucleotide sequence 5′-CTTTGWW-3’ through a single HMG domain. Despite their similarity, the LEF1 position weight matrices did not correspond, in general, to the SOX2 matrix given no bias towards C before the first position in the motif or towards a T at position two was detected.

The genomic regions with epigenetic characteristics of active enhancers in both quiescent and actively dividing NSCs are thus enriched in TCF/LEF1 transcription factor DNA-binding motifs. In enhancers that are specifically active in the quiescent state, by *de novo* motif searches we found putative heterotrimeric transcription factor (NFYA, NFYB) motifs and the nuclear factor 1 dimer (NFIA and NFIX) motifs flanking the LEF1 binding sites, although it remains to be explored if there is a bias in positioning. We further show that LEF1 is able to interact with NFIX in a heterologous system. Whether both factors interact at the quiescence-specific enhancers to regulate nearby genes in NSPCs warrants further investigation. So far the analysis performed identified 115 candidate genes upregulated in quiescence showing quiescence-specific enhancers with putative LEF1 and NFIX target sites, including the lysophosphatidic acid receptor (LPA_1_) gene (*Lpar1*), a recently described adult hippocampal NSPC marker (Walker et al., 2016). LPA is a versatile bioactive phospholipid with diverse biological functions during development of the nervous system (Geraldo et al., 2021). LPA is also interesting given lipids are emerging as key regulators of adult neural stem cell decisions (Clémot et al., 2020; Lee et al., 2020). NSPCs can be isolated from the adult hippocampal DG taking advantage of LPA_1_-GFP transgenic mice, yet only a subset of the precursors are proliferative cells based on the surface expression of other markers such as EGFR and Prominin-1. The remainder (majority) of LPA_1_-GFP are classified as non-proliferative or quiescent precursor cells (Walker et al., 2016; Valcárcel-Martín et al., 2020), so it is tempting to speculate that LEF1 may be involved in maintaining the expression of *Lpar1* in quiescent NSPCs. Finally, it is worth to note that the Martynoga et al. (2013) study (on which the analysis of LEF1 binding sites in enhancers is based) was performed in mouse NSPCs expanded in FGF2 and EGF, while our study employed rat NSPCs expanded in FGF2 only. Consequently, the upregulation of a subset of shared LEF1 target genes among the two studies may point to a conserved mechanism relevant to the regulation Q-NSPCs in different species and growth conditions.

## Conclusion

In summary, our results highlight the role of LEF1 in the transactivation of genes not only in active NSPCs as expected, but also in quiescent NSPCs. Furthermore, we show that alternative splicing changes the preponderance of different LEF1 isoforms at the transition from activation to quiescence. LEF1 E6 inclusion is favoured in quiescent cells while E6 skipping predominates in actively proliferating cells. This post-transcriptional regulatory event adds up to other post-transcriptional control points of key transcription factors recently identified in quiescent stem cells. In this line, in adult hippocampal NSCs it has been reported that, in order to return to the quiescent state, the pro-activation transcription factor ASCL1 is destabilized by the E3-ubiquitin ligase Huwe1 (Urbán et al., 2016). The degradation of ASCL1 is further promoted by Id4 expressed in quiescent radial glia-like NSCs (Blomfield et al., 2019; Zhang et al., 2019), while in muscle stem cells MyoD is transcribed during quiescence yet its translation is inhibited by an RNA-binding protein (de Morrée et al., 2017). A common emerging theme, therefore, is that transcription factors involved in shaping the transcriptional landscape of active and quiescent adult stem cells require fine post-transcriptional regulation to fulfill their function. An interesting field to explore in the future is the possible differential regulatory function of the long and short LEF1 isoforms, beyond the implications on protein stability discovered in the current study. Proteomic studies will be needed to reveal whether changes in the CRD are relevant for the cooperation of LEF1 with other transcription factors and the organization of the transcriptional landscape that determines the transition between quiescence and activation.

## Data Availability

The datasets generated during the current study are available in the gene expression omnibus GEO dataset repository (GSE158658).

## References

[B1] Aloysius A. DasGupta R. Dhawan J. (2018). The transcription factor Lef1 switches partners from β-catenin to Smad3 during muscle stem cell quiescence. Sci. Signal. 11, eaan3000. 10.1126/scisignal.aan3000 30042129

[B2] Amador-Arjona A. Cimadamore F. Huang C.-T. Wright R. Lewis S. Gage F. H. (2015). SOX2 primes the epigenetic landscape in neural precursors enabling proper gene activation during hippocampal neurogenesis. Proc. Natl. Acad. Sci. U.S.A. 112, E1936–E1945. 10.1073/pnas.1421480112 25825708 PMC4403144

[B3] Andersen J. Urbán N. Achimastou A. Ito A. Simic M. Ullom K. (2014). A transcriptional mechanism integrating inputs from extracellular signals to activate hippocampal stem cells. Neuron 83, 1085–1097. 10.1016/j.neuron.2014.08.004 25189209 PMC4157576

[B4] Arce L. Yokoyama N. N. Waterman M. L. (2006). Diversity of LEF/TCF action in development and disease. Oncogene 25, 7492–7504. 10.1038/sj.onc.1210056 17143293

[B5] Armenteros T. Andreu Z. Hortigüela R. Lie D. C. Mira H. (2018). BMP and WNT signalling cooperate through LEF1 in the neuronal specification of adult hippocampal neural stem and progenitor cells. Sci. Rep. 8, 9241. 10.1038/s41598-018-27581-0 29915186 PMC6006330

[B6] Arredondo S. B. Guerrero F. G. Herrera-Soto A. Jensen-Flores J. Bustamante D. B. Oñate-Ponce A. (2020). Wnt5a promotes differentiation and development of adult-born neurons in the hippocampus by noncanonical Wnt signaling. Stem Cells 38, 422–436. 10.1002/stem.3121 31721364

[B7] Austin S. H. L. Gabarró-Solanas R. Rigo P. Paun O. Harris L. Guillemot F. (2021). Wnt/beta-catenin signalling is dispensable for adult neural stem cell homeostasis and activation. Development 148, dev199629. 10.1242/dev.199629 34557919 PMC8572000

[B8] Bailey T. L. (2011). DREME: motif discovery in transcription factor ChIP-seq data. Bioinformatics 27, 1653–1659. 10.1093/bioinformatics/btr261 21543442 PMC3106199

[B9] Berg D. A. Su Y. Jimenez-Cyrus D. Patel A. Huang N. Morizet D. (2019). A common embryonic origin of stem cells drives developmental and adult neurogenesis. Cell 177, 654–668. 10.1016/j.cell.2019.02.010 30929900 PMC6496946

[B10] Blomfield I. M. Rocamonde B. Masdeu M. D. M. Mulugeta E. Vaga S. van den Berg D. L. (2019). Id4 promotes the elimination of the pro-activation factor Ascl1 to maintain quiescence of adult hippocampal stem cells. Elife 8, e48561. 10.7554/eLife.48561 31552825 PMC6805120

[B11] Choe Y. Kozlova A. Graf D. Pleasure S. J. (2013). Bone morphogenic protein signaling is a major determinant of dentate development. J. Neurosci. 33, 6766–6775. 10.1523/JNEUROSCI.0128-13.2013 23595735 PMC3684166

[B12] Choe Y. Pleasure S. J. Mira H. (2015). Control of adult neurogenesis by short-range morphogenic-signaling molecules. Cold Spring Harb. Perspect. Biol. 8, a018887. 10.1101/cshperspect.a018887 26637286 PMC4772099

[B13] Clémot M. Sênos Demarco R. Jones D. L. (2020). Lipid mediated regulation of adult stem cell behavior. Front. Cell Dev. Biol. 8, 115. 10.3389/fcell.2020.00115 32185173 PMC7058546

[B14] Clevers H. Nusse R. (2012). Wnt/β-Catenin signaling and disease. Cell 149, 1192–1205. 10.1016/j.cell.2012.05.012 22682243

[B15] Clevers H. Loh K. M. Nusse R. (2014). Stem cell signaling. An integral program for tissue renewal and regeneration: Wnt signaling and stem cell control. Science 346, 1248012. 10.1126/science.1248012 25278615

[B16] Conti L. Pollard S. M. Gorba T. Reitano E. Toselli M. Biella G. (2005). Niche-independent symmetrical self-renewal of a mammalian tissue stem cell. PLoS Biol. 3, e283. 10.1371/journal.pbio.0030283 16086633 PMC1184591

[B17] de Morrée A. van Velthoven C. T. J. Gan Q. Salvi J. S. Klein J. D. D. Akimenko I. (2017). Staufen1 inhibits MyoD translation to actively maintain muscle stem cell quiescence. Proc. Natl. Acad. Sci. U.S.A. 114, E8996. 10.1073/pnas.1708725114 29073096 PMC5664522

[B18] DeCarolis N. A. Mechanic M. Petrik D. Carlton A. Ables J. L. Malhotra S. (2013). In vivocontribution of nestin- and GLAST-lineage cells to adult hippocampal neurogenesis. Hippocampus 23, 708–719. 10.1002/hipo.22130 23554226 PMC3732558

[B19] Fuerer C. Nusse R. (2010). Lentiviral vectors to probe and manipulate the Wnt signaling pathway. PLoS One 5, e9370. 10.1371/journal.pone.0009370 20186325 PMC2826402

[B20] Galceran J. Miyashita-Lin E. M. Devaney E. Rubenstein J. L. Grosschedl R. (2000). Hippocampus development and generation of dentate gyrus granule cells is regulated by LEF1. Development 127, 469–482. 10.1242/dev.127.3.469 10631168

[B21] Gao Z. Ure K. Ables J. L. Lagace D. C. Nave K.-A. Goebbels S. (2009). Neurod1 is essential for the survival and maturation of adult-born neurons. Nat. Neurosci. 12, 1090–1092. 10.1038/nn.2385 19701197 PMC3365543

[B22] Geraldo L. H. M. Spohr T. C. L. d. S. Amaral R. F. d. Fonseca A. C. C. d. Garcia F. d. A. Freitas C. (2021). Role of lysophosphatidic acid and its receptors in health and disease: novel therapeutic strategies. Sig Transduct. Target Ther. 6, 45. 10.1038/s41392-020-00367-5 PMC7851145 33526777

[B23] Ghogomu S. M. van Venrooy S. Ritthaler M. Wedlich D. Gradl D. (2006). HIC-5 is a novel repressor of lymphoid enhancer factor/T-cell factor-driven transcription. J. Biol. Chem. 281, 1755–1764. 10.1074/jbc.M505869200 16291758

[B24] Gonçalves J. T. Schafer S. T. Gage F. H. (2016). Adult neurogenesis in the hippocampus: From stem cells to behavior. Cell 167, 897–914. 10.1016/j.cell.2016.10.021 27814520

[B25] Gupta S. Stamatoyannopoulos J. A. Bailey T. L. Noble W. (2007). Quantifying similarity between motifs. Genome Biol. 8, R24. 10.1186/gb-2007-8-2-r24 17324271 PMC1852410

[B26] Harris L. Rigo P. Stiehl T. Gaber Z. B. Austin S. H. L. Masdeu M. D. M. (2021). Coordinated changes in cellular behavior ensure the lifelong maintenance of the hippocampal stem cell population. Cell Stem Cell 28, 863–876. 10.1016/j.stem.2021.01.003 33581058 PMC8110946

[B27] Haslinger A. Schwarz T. J. Covic M. Chichung Lie D. (2009). Expression of Sox11 in adult neurogenic niches suggests a stage-specific role in adult neurogenesis. Eur. J. Neurosci. 29, 2103–2114. 10.1111/j.1460-9568.2009.06768.x 19490090

[B28] Heppt J. Wittmann M. T. Schäffner I. Billmann C. Zhang J. Vogt Weisenhorn D. (2020). β catenin signaling modulates the tempo of dendritic growth of adult born hippocampal neurons. EMBO J. 39, e104472. 10.15252/embj.2020104472 32929771 PMC7604596

[B29] Hikasa H. Sokol S. Y. (2011). Phosphorylation of TCF proteins by homeodomain-interacting protein kinase 2. J. Biol. Chem. 286, 12093–12100. 10.1074/jbc.M110.185280 21285352 PMC3069413

[B30] Hikasa H. Ezan J. Itoh K. Li X. Klymkowsky M. W. Sokol S. Y. (2010). Regulation of TCF3 by Wnt-dependent phosphorylation during vertebrate axis specification. Dev. Cell 19, 521–532. 10.1016/j.devcel.2010.09.005 20951344 PMC2963175

[B31] Hodge R. D. Nelson B. R. Kahoud R. J. Yang R. Mussar K. E. Reiner S. L. (2012). Tbr2 is essential for hippocampal lineage progression from neural stem cells to intermediate progenitors and neurons. J. Neurosci. 32, 6275–6287. 10.1523/JNEUROSCI.0532-12.2012 22553033 PMC3366485

[B32] Hovanes K. Li T. W. H. Munguia J. E. Truong T. Milovanovic T. Lawrence Marsh J. (2001). β-catenin-sensitive isoforms of lymphoid enhancer factor-1 are selectively expressed in colon cancer. Nat. Genet. 28, 53–57. 10.1038/ng0501-53 11326276

[B33] Ishitani T. Ninomiya-Tsuji J. Matsumoto K. (2003). Regulation of lymphoid enhancer factor 1/T-cell factor by mitogen-activated protein kinase-related nemo-like kinase-dependent phosphorylation in wnt/β-catenin signaling. Mol. Cell. Biol. 23, 1379–1389. 10.1128/MCB.23.4.1379-1389.2003 12556497 PMC141159

[B34] Itasaki N. Hoppler S. (2010). Crosstalk between Wnt and bone morphogenic protein signaling: A turbulent relationship. Dev. Dyn. 239, 16. 10.1002/dvdy.22009 19544585

[B35] Jesse S. Koenig A. Ellenrieder V. Menke A. (2010). Lef-1 isoforms regulate different target genes and reduce cellular adhesion. Int. J. Cancer 126, 1109. 10.1002/ijc.24802 19653274

[B36] Knobloch M. Pilz G.-A. Ghesquière B. Kovacs W. J. Wegleiter T. Moore D. L. (2017). A fatty acid oxidation-dependent metabolic shift regulates adult neural stem cell activity. Cell Rep. 20, 2144–2155. 10.1016/j.celrep.2017.08.029 28854364 PMC5583518

[B37] Kuwabara T. Hsieh J. Muotri A. Yeo G. Warashina M. Lie D. C. (2009). Wnt-mediated activation of NeuroD1 and retro-elements during adult neurogenesis. Nat. Neurosci. 12, 1097–1105. 10.1038/nn.2360 19701198 PMC2764260

[B38] Lee D. Kim Y. H. Kim J. H. (2020). The role of lysophosphatidic acid in adult stem cells. Int. J. Stem Cells 13, 182–191. 10.15283/ijsc20035 32587135 PMC7378901

[B39] Leeman D. S. Hebestreit K. Ruetz T. Webb A. E. McKay A. Pollina E. A. (2018). Lysosome activation clears aggregates and enhances quiescent neural stem cell activation during aging. Science 359, 1277–1283. 10.1126/science.aag3048 29590078 PMC5915358

[B40] Li L. Medina-Menéndez C. García-Corzo L. Córdoba-Beldad C. M. Quiroga A. C. Calleja Barca E. (2022). SoxD genes are required for adult neural stem cell activation. Cell Rep. 38, 110313. 10.1016/j.celrep.2022.110313 35108528 PMC11783645

[B41] Lie D.-C. Colamarino S. A. Song H.-J. Désiré L. Mira H. Consiglio A. (2005). Wnt signalling regulates adult hippocampal neurogenesis. Nature 437, 1370–1375. 10.1038/nature04108 16251967

[B42] Livak K. J. Schmittgen T. D. (2001). Analysis of relative gene expression data using real-time quantitative PCR and the 2-ΔΔCT method. Methods 25, 402–408. 10.1006/meth.2001.1262 11846609

[B43] Llorens-Bobadilla E. Zhao S. Baser A. Saiz-Castro G. Zwadlo K. Martin-Villalba A. (2015). Single-cell transcriptomics reveals a population of dormant neural stem cells that become activated upon brain injury. Cell Stem Cell 17, 329–340. 10.1016/j.stem.2015.07.002 26235341

[B44] Lustig B. Jerchow B. Sachs M. Weiler S. Pietsch T. Karsten U. (2002). Negative feedback loop of Wnt signaling through upregulation of conductin/axin2 in colorectal and liver tumors. Mol. Cell. Biol. 22, 1184–1193. 10.1128/mcb.22.4.1184-1193.2002 11809809 PMC134640

[B45] Mallory M. J. Jackson J. Weber B. Chi A. Heyd F. Lynch K. W. (2011). Signal- and development-dependent alternative splicing of LEF1 in T cells is controlled by CELF2. Mol. Cell. Biol. 31, 2184–2195. 10.1128/MCB.05170-11 21444716 PMC3133246

[B46] Maretto S. Cordenonsi M. Dupont S. Braghetta P. Broccoli V. Hassan A. B. (2003). Mapping Wnt/β-catenin signaling during mouse development and in colorectal tumors. Proc. Natl. Acad. Sci. U.S.A. 100, 3299–3304. 10.1073/pnas.0434590100 12626757 PMC152286

[B47] Marqués-Torrejón M. Á. Williams C. A. C. Southgate B. Alfazema N. Clements M. P. Garcia-Diaz C. (2021). LRIG1 is a gatekeeper to exit from quiescence in adult neural stem cells. Nat. Commun. 12, 2594. 10.1038/s41467-021-22813-w 33972529 PMC8110534

[B48] Martynoga B. Mateo J. L. Zhou B. Andersen J. Achimastou A. Urbán N. (2013). Epigenomic enhancer annotation reveals a key role for NFIX in neural stem cell quiescence. Genes Dev. 27, 1769–1786. 10.1101/gad.216804.113 23964093 PMC3759694

[B49] Matsuda S. Kuwako K.-i. Okano H. J. Tsutsumi S. Aburatani H. Saga Y. (2012). Sox21 promotes hippocampal adult neurogenesis via the transcriptional repression of the Hes5 gene. J. Neurosci. 32, 12543–12557. 10.1523/JNEUROSCI.5803-11.2012 22956844 PMC6621257

[B50] Michaelidis T. M. Lie D. C. (2008). Wnt signaling and neural stem cells: caught in the Wnt web. Cell Tissue Res. 331, 193–210. 10.1007/s00441-007-0476-5 17828608

[B51] Mira H. Andreu Z. Suh H. Lie D. C. Jessberger S. Consiglio A. (2010). Signaling through BMPR-IA regulates quiescence and long-term activity of neural stem cells in the adult hippocampus. Cell Stem Cell 7, 78–89. 10.1016/j.stem.2010.04.016 20621052

[B52] Morales A. V. Mira H. (2019). Adult neural stem cells: born to last. Front. Cell Dev. Biol. 7, 96. 10.3389/fcell.2019.00096 31214589 PMC6557982

[B53] Morizur L. Chicheportiche A. Gauthier L. R. Daynac M. Boussin F. D. Mouthon M.-A. (2018). Distinct molecular signatures of quiescent and activated adult neural stem cells reveal specific interactions with their microenvironment. Stem Cell Rep. 11, 565–577. 10.1016/j.stemcr.2018.06.005 PMC6092681 29983386

[B54] Moss J. Gebara E. Bushong E. A. Sánchez-Pascual I. O’Laoi R. El M’Ghari I. (2016). Fine processes of Nestin-GFP-positive radial glia-like stem cells in the adult dentate gyrus ensheathe local synapses and vasculature. Proc. Natl. Acad. Sci. U.S.A. 113, E2536–E2545. 10.1073/pnas.1514652113 27091993 PMC4983830

[B55] Mu L. Berti L. Masserdotti G. Covic M. Michaelidis T. M. Doberauer K. (2012). SoxC transcription factors are required for neuronal differentiation in adult hippocampal neurogenesis. J. Neurosci. 32, 3067–3080. 10.1523/JNEUROSCI.4679-11.2012 22378879 PMC3356877

[B56] Mukherjee S. Brulet R. Zhang L. Hsieh J. (2016). REST regulation of gene networks in adult neural stem cells. Nat. Commun. 7, 13360. 10.1038/ncomms13360 27819263 PMC5103073

[B57] Muñoz Descalzo S. Martinez Arias A. (2012). The structure of Wntch signalling and the resolution of transition states in development. Seminars Cell Dev. Biol. 23, 443–449. 10.1016/j.semcdb.2012.01.012 22326376

[B58] Muñoz-Cánoves P. Huch M. (2018). Definitions for adult stem cells debated. Nature 563, 328–329. 10.1038/d41586-018-07175-6 30378594

[B59] Nagalski A. Irimia M. Szewczyk L. Ferran J. L. Misztal K. Kuznicki J. (2013). Postnatal isoform switch and protein localization of LEF1 and TCF7L2 transcription factors in cortical, thalamic, and mesencephalic regions of the adult mouse brain. Brain Struct. Funct. 218, 1531–1549. 10.1007/s00429-012-0474-6 23152144 PMC3825142

[B60] Ortiz‐Matamoros A. Arias C. (2019). Differential changes in the number and morphology of the new neurons after chronic infusion of Wnt7a, Wnt5a, and dkk 1 in the adult Hippocampus *in vivo* . Anat. Rec. 302, 1647–1657. 10.1002/ar.24069 30635974

[B61] Ozen I. Galichet C. Watts C. Parras C. Guillemot F. Raineteau O. (2007). Proliferating neuronal progenitors in the postnatal hippocampus transiently express the proneural gene Ngn2. Eur. J. Neurosci. 25, 2591–2603. 10.1111/j.1460-9568.2007.05541.x 17466019

[B62] Palmer T. D. Takahashi J. Gage F. H. (1997). The adult rat hippocampus contains primordial neural stem cells. Mol. Cell. Neurosci. 8, 389–404. 10.1006/mcne.1996.0595 9143557

[B63] Qu Q. Sun G. Murai K. Ye P. Li W. Asuelime G. (2013). Wnt7a regulates multiple steps of neurogenesis. Mol. Cell. Biol. 33, 2551–2559. 10.1128/MCB.00325-13 23629626 PMC3700117

[B64] Rodgers J. T. Schroeder M. D. Ma C. Rando T. A. (2017). HGFA is an injury-regulated systemic factor that induces the transition of stem cells into G alert. Cell Rep. 19, 479–486. 10.1016/j.celrep.2017.03.066 28423312 PMC5468096

[B65] Roybon L. Hjalt T. Stott S. Guillemot F. Li J.-Y. Brundin P. (2009). Neurogenin2 directs granule neuroblast production and amplification while NeuroD1 specifies neuronal fate during hippocampal neurogenesis. PLoS One 4, e4779. 10.1371/journal.pone.0004779 19274100 PMC2652712

[B66] Schafer S. T. Han J. Pena M. von Bohlen Und Halbach O. Peters J. Gage F. H. (2015). The Wnt adaptor protein ATP6AP2 regulates multiple stages of adult hippocampal neurogenesis. J. Neurosci. 35, 4983–4998. 10.1523/JNEUROSCI.4130-14.2015 25810528 PMC4389597

[B67] Shin J. Berg D. A. Zhu Y. Shin J. Y. Song J. Bonaguidi M. A. (2015). Single-cell RNA-seq with waterfall reveals molecular cascades underlying adult neurogenesis. Cell Stem Cell 17, 360–372. 10.1016/j.stem.2015.07.013 26299571 PMC8638014

[B68] Subramaniam S. Sreenivas P. Cheedipudi S. Reddy V. R. Shashidhara L. S. Chilukoti R. K. (2014). Distinct transcriptional networks in quiescent myoblasts: a role for Wnt signaling in reversible vs. irreversible arrest. PLoS One 8, e65097. 10.1371/journal.pone.0065097 23755177 PMC3670900

[B69] Sueda R. Imayoshi I. Harima Y. Kageyama R. (2019). High Hes1 expression and resultant Ascl1 suppression regulate quiescent vs. active neural stem cells in the adult mouse brain. Genes Dev. 33, 511–523. 10.1101/gad.323196.118 30862661 PMC6499325

[B70] Sun Y. Hu J. Zhou L. Pollard S. M. Smith A. (2011). Interplay between FGF2 and BMP controls the self-renewal, dormancy and differentiation of rat neural stem cells. J. Cell Sci. 124, 1867–1877. 10.1242/jcs.085506 21558414 PMC3096055

[B71] Tapial J. Ha K. C. H. Sterne-Weiler T. Gohr A. Braunschweig U. Hermoso-Pulido A. (2017). An atlas of alternative splicing profiles and functional associations reveals new regulatory programs and genes that simultaneously express multiple major isoforms. Genome Res. 27, 1759–1768. 10.1101/gr.220962.117 28855263 PMC5630039

[B72] Urbán N. van den Berg D. L. C. Forget A. Andersen J. Demmers J. A. A. Hunt C. (2016). Return to quiescence of mouse neural stem cells by degradation of a proactivation protein. Science 353, 292–295. 10.1126/science.aaf4802 27418510 PMC5321528

[B73] Valcárcel-Martín R. Martín-Suárez S. Muro-García T. Pastor-Alonso O. Rodríguez de Fonseca F. Estivill-Torrús G. (2020). Lysophosphatidic acid receptor 1 specifically labels seizure-induced hippocampal reactive neural stem cells and regulates their division. Front. Neurosci. 14, 811. 10.3389/fnins.2020.00811 32922255 PMC7456947

[B74] van Velthoven C. T. J. Rando T. A. (2019). Stem cell quiescence: Dynamism, restraint, and cellular idling. Cell Stem Cell 24, 213–225. 10.1016/j.stem.2019.01.001 30735649 PMC6413865

[B75] Varela-Nallar L. Inestrosa N. C. (2013). Wnt signaling in the regulation of adult hippocampal neurogenesis. Front. Cell. Neurosci. 7, 100. 10.3389/fncel.2013.00100 23805076 PMC3693081

[B76] Walker T. L. Overall R. W. Vogler S. Sykes A. M. Ruhwald S. Lasse D. (2016). Lysophosphatidic acid receptor is a functional marker of adult hippocampal precursor cells. Stem Cell Rep. 6, 552–565. 10.1016/j.stemcr.2016.03.002 PMC4834054 27050949

[B77] Yamada M. Ohnishi J. Ohkawara B. Iemura S. Satoh K. Hyodo-Miura J. (2006). NARF, an nemo-like kinase (NLK)-associated ring finger protein regulates the ubiquitylation and degradation of T cell factor/lymphoid enhancer factor (TCF/LEF). J. Biol. Chem. 281, 20749–20760. 10.1074/jbc.M602089200 16714285

[B78] Zhang R. Boareto M. Engler A. Louvi A. Giachino C. Iber D. (2019). Id4 downstream of notch2 maintains neural stem cell quiescence in the adult hippocampus. Cell Rep. 28, 1485–1498. 10.1016/j.celrep.2019.07.014 31390563

[B79] Zhou C.-J. Zhao C. Pleasure S. J. (2004). Wnt signaling mutants have decreased dentate granule cell production and radial glial scaffolding abnormalities. J. Neurosci. 24, 121–126. 10.1523/JNEUROSCI.4071-03.2004 14715945 PMC6729560

