# Supplementary material for: The transcription factor LEF1 interacts with NFIX and switches isoforms during adult hippocampal neural stem cell quiescence
Source: Front Cell Dev Biol. 2022 Jul 22;10:912319. doi: 10.3389/fcell.2022.912319 (PMC9355129; doi:10.3389/fcell.2022.912319)
Supplement: Supplementary file 4 [file DataSheet1.pdf]

Supplementary Material

Materials and Methods

Animals

WT C57BL/6JRccHsd mice (P3-P21 days-old) were obtained from in-house breeding. Mice were maintained under specific-pathogen-free conditions and all manipulations were approved by the Ethics Committee of the Spanish National Research Council, Spain. Animal experimentation followed the recommendations of the Federation of European Laboratory Animal Science Associations on health monitoring, European Community Law (2010/63/UE), and Spanish law (R.D. 53/2013).

GLAST$^+$ hippocampal cell isolation

Mice were anesthetized and decapitated. Brains were removed and hippocampi were dissected from 3 and 21-days-old mice and dissociated using the NTDK (T) in combination with the gentleMACS Octo Dissociator (both from Miltenyi Biotec). Cell suspensions containing $1x10^7$ cells were labelled with magnetic MicroBeads coupled to antibodies specific for the radial glia and astrocyte marker GLAST (Anti-GLAST (ACSA-1) MicroBead Kit, mouse; Miltenyi Biotec). Cells were suspended in 500 µL of PBS with 0.5% BSA and the cell suspension was loaded onto a MS Column (Miltenyi Biotec), which was placed in the magnetic field of a MiniMACS™ Separator (Miltenyi Biotec). The magnetically labelled GLAST-positive cells were retained within the column and eluted as the positively selected cell fraction after removing the column from the magnet. Cell number was determined and GLAST$^+$ cells were then used for immunocytochemistry assays or for total RNA extraction followed by RT-qPCR or RT-PCR. GLAST$^+$ cells were seeded onto Matrigel®-coated glass coverslips and maintained in a humidified atmosphere (5% CO_2, 95% air) at 37ºC for 1 hour in N2 medium. Cultures cells were fixed with 2% PFA in PBS.

Cell culture

Adult rat hippocampal neural stem and progenitor cells (NSPCs) were grown in N2 medium (DMEM/F-12 (1:1) adding N2 Supplement (1x) (Gibco). NSPCs were cultured as neurospheres with 10 ng/mL of fibroblast growth factor 2 (FGF-2) (PeproTech) in the proliferation state and with 10ng/mL of FGF2 and 50ng/mL of bone morphogenetic protein 4 (BMP4) (PeproTech) to induce the quiescent state for four days *in vitro* (DIV). For differentiation assays NSPCs were cultured in MW24 dishes using poly-ornitine/laminin treated 12 mm cover glasses (ThermoScientific), using 1 µM Retinoic Acid (Sigma-Aldrich) and 5 µM Forskolin (Sigma-Aldrich). For functional assays, CHIR 99021 (Sigma Aldrich) was added at the indicated concentration. The isolation, characterization and culturing of adult rat hippocampal neural stem and progenitor cells (NSPCs) used in this study have been described in Palmer et al., 1997. Neuro2A cells were grown in DMEM medium supplemented with 10% Fetal Bovine Serum (FBS) and 1% antibiotics.

Microarray

Total RNA was extracted from Q- and A-NSPCs (Qiagen). Six RNA samples were analysed, since RNA was isolated from proliferating NSPCs grown in FGF-2 and quiescent NSPCs grown in FGF-2+BMP4, 3 biological replicates for each condition. Gene expression analysis by microarrays was performed with Affymetrix® GeneChip® technology. All steps of the

process were carried out with equipment and protocols recommended by Affymetrix. The Ambion WT Expression kit, combined with the GeneChip® WT Terminal Labeling and Controls Kit (Affymetrix) was used to generate amplified and biotinylated sense strand DNA targets from the entire expressed genome. The software used for data analysis was Affymetrix® GeneChip® Command Console® (AGCC 3.1, Affymetrix®) and Expression Console™ (EC 1.1, Affymetrix®). Detailed information about the GeneChip technology and Rat Gene 1.1 ST used in this study can be found on the Affymetrix web page. dChip (www.dchip.org) (Li and Wong, 2001) and Partek Genomics Suite (www.partek.com) were used for the bioinformatic analysis. Data was deposited in the gene expression omnibus GEO dataset (GSE158658).

Estimation of exon retention

Efficiency of exon splicing was calculated by standard RT-PCR. Primers were designed at the boundaries of each *Lef1* exon (Supplementary Table 4a) and PCR was performed employing cDNA obtained from Q- and A-NSPCs as template. The % exon retention was calculated using *IS*100/(IS + ES)* where *IS (inclusion signal)* corresponds to the intensity of the PCR band in gel electrophoresis that includes the exon and *ES (exclusion signal)* is the intensity of the band that excludes the exon. Values indicate the percentage of transcripts that contain the exon compared to the total transcript population.

RT- qPCR

RNA was extracted from proliferating and quiescent cells collected at 4 DIV using Illustra RNAspin Mini Kit (GE Healthcare). cDNA was obtained by reverse-transcription (RT) employing PrimeScript RT Reagent Kit (Takara). Gene expression was determined by quantitative PCR (qPCR) or standard PCR using TB Green Premix EX Taq (2×)(Takara) and the corresponding forward and reverse primer for each gene are listed in Supplementary Table 4b. *Sdha* was used as the internal reference for normalization. Data were analysed according to the $2^{-\Delta\Delta Ct}$ method (Livak and Schmittgen, 2001).

Supplementary Table 4a. List of primers sequences used for conventional PCR and PCR amplicon sizes for PSI calculation.

Primer	***Sequence (5’-3’)***	***PCR amplicon corresponding to exon inclusion (bp)***	***PCR amplicon corresponding to exon exclusion (bp)***
Lef1 Ex1 Fw<br>Lef1 Ex1 Rv	CGGAGATTACAGAGCCGCCGGGAT<br>GCCTTGTCGTGGTAGGGCTCCTGA	275	68
Lef1 Ex2 Fw<br>Lef1 Ex2 Rv	CGAAGAGGAGGGCGACTTAG<br>GGGTCGCTATTCATATTGGGC	240	173
Lef1 Ex3 Fw<br>Lef1 Ex3 Rv	GAGCCCTACCACGACAAGG<br>CTGTACGTGATGAGGGGCG	242	108
Lef1 Ex4 Fw<br>Lef1 Ex4 Rv	GCCCAATATGAATAGCGACCC<br>GGGGTAGAAGGTGGGCATTT	244	111
Lef1 Ex5 Fw	CGACGAGCACTTTTCTCCAG		

Lef1 Ex5 Rv	GATGGGTAGGCTTGCCTGAA	165	119
Lef1 Ex6 Fw<br>Lef1 Ex6 Rv	ACCCTCCAGCTCCTGAAATGC<br>TTAGGTCGCTGTCCGTGTGG	279	195
Lef1 Ex7 Fw<br>Lef1 Ex7 Rv	GCCTGTTTATCCCATCACGG<br>TGAGGTCTTTTAGGCTCCTGT	246	123
Lef1 Ex8 Fw<br>Lef1 Ex8 Rv	ACACAACTGGCATCCCTCAT<br>TATTTGGCCTGCTCTTCCCG	283	120
Lef1 Ex9 Fw<br>Lef1 Ex9 Rv	AGCCGAGTGTACGCTAAAGG<br>TCCTGTAGCTTCTCTCTCTTCC	201	93
Lef1 Ex10 Fw<br>Lef1 Ex10 Rv	CGGGAAGAGCAGGCCAAATA<br>TAGGCAGCTGTCATTCTGGG	170	121
Lef1 Ex11 Fw<br>Lef1 Ex11 Rv	CGCGAGACAATTATGGCAAG<br>TTCAACAAGCTTCCATCTCCA	184	149
Lef1 Ex12 Fw<br>Lef1 Ex12 Rv	CAGCTGCCTACATCTGAAACATG<br>CTAGCAGTGACCTCAGGGTAAA	182	132

Supplementary Table 4b. List of primers sequences used for qPCR.

Primer	***Sequence (5'-3')***
Lef1 Fw<br>Lef1 Rv	GATCCCACCCGTCACACATC<br>AGGTGGGGTGATCTGTCCAAC
Fzd8 Fw<br>Fzd8 Rv	GCTCTACAACCGCGTCAAGAC<br>CGGGTACTTAAAGCGCTCCAT
Gsk3b Fw<br>Gsk3b Rv	ACCAAATGGGCGAGACACA<br>GGTCTGTCCACGGTCTCCA
Id1 Fw<br>Id1 Rv	ACGGCGAGATCAGTGCCTT<br>CCTCCTGAAGGGCTGGAGTC
Lpar1 Fw<br>Lpar1 Rv	GCATAGTTCTGGACCCAGGAGGAA<br>CCGGAGTCCAGCAGACAATAAAGG
Acox1 Fw<br>Acox1 Rv	CTTCGAGGCTTGGAAACCAC<br>AAAGGCATGTAACCCGTAGCA
Abcc1 Fw<br>Abcc1Rv	AAACTGTCCGTGACCCGAATCCAT<br>GCACCATCATCCCTGTAATCCAC
Trim2 Fw<br>Trim2 Rv	ACTTTCTGCGAGAGGTGCCTGCA<br>TCACATTTCCATCGTGGTTCGGGC
mCcherry Fw<br>mCherry Rv	GAACGGCCACGAGTTCGAGA<br>CTTGGAGCCGTACATGAACTGAGG
GFP Fw<br>GFP Rv	AAGCAGCACGACTTCTTCAAGTC<br>AGACGTTGTGGCTGTTGTAGTTGT

Western Blot

Cells were lysed and fractionated by SDS-PAGE. Membranes were incubated with 5% non-fat milk or 5% BSA (Sigma) in T-TBS. After incubation with primary and secondary antibodies, membranes were visualized with ECL, analysed with Fiji Program and normalised against the corresponding intensity of β-actin. The following antibodies were used as primary antibodies: Lef1 (Cell Signalling, #2230, 1:200), Lef1 alternate exon (Exalpha, X1075M, 1:200), β-actin (Sigma, A5441, 1:5000), Tubulin alpha (Sigma, T9026, 1:5000), β-catenin non-phosphorylated (Cell Signaling, 8814, 1:500), HA-tag (Sigma Aldrich, H3663, 1:2000) and GFP (Abcam, ab290, 1:1000). Secondary antibodies were peroxidase-conjugated goat anti-rabbit IgG (Jackson ImmunoResearch, 111-035-003) and peroxidase-conjugated goat anti-mouse (Jackson ImmunoResearch, 115-035-003). For half-life experiments, cells were incubated with cycloheximide (CHx; 100 mg/ml, Sigma Aldrich) and lysed at different times.

Protein dephosphorylation assay

Proteins were dephosphorylated before SDS-PAGE. Q- and A-NSPCs cells were lysed in the presence of protease inhibitors and crude extracts were treated with 1U of alkaline phosphatase (Thermo scientific, #EF0654) at 37ºC for one hour. To stop reaction EDTA 0.5M was added.

Immunocytochemistry

NSPCs and GLAST$^+$ cells were fixed with 2% paraformaldehyde (PFA). Cells were blocked with TB (10% FBS, 0.5% Triton-X100), then incubated with different primary antibody solutions overnight at 4ºC. Secondary antibodies were incubated for one hour. After staining, all sections and cells were mounted and preserved with 50% Mowiol 4-88 (Roth, 0718.2), 2.5% DABCO (Roth, 0713.2). Images were obtained with Leica Spectral SP8 confocal microscope with a 40x oil objective. Images were analysed with Image J Fiji software. Primary antibodies were: β-tubulin (Tuj1) (Covance, MMS-435P, 1:200), Ki67 (Abcam, ab15580, 1:150) and BrdU (Abcam, ab6326, 1:200).

Lentiviral production

Lentiviral vector 7xTCF-eGFP-SV40-mCherry (p7GC) was produced as below. HEK293T cells were co-transfected with p7TGC along with pMD2.G (Plasmid #12259, Addgene) and psPAX2 (Plasmid #12260, Addgene) by Calcium-Phosphate precipitation. The medium was replaced after 8 hours and N2 medium was conditioned twice for 24 hours. The media containing the viral particles was pooled, filtered through 0.45 µm PES filter and concentrated by Vivaspin® 20 ultrafiltration unit (Sartorious, VS2042). The concentrated virus suspension was aliquoted and stored at -80ºC. p7GC vector was kindly provided by Purificación Muñoz, IDIBELL, Barcelona.

Wnt reporter assays

For the 7xTCF-eGFP-SV40-mCherry (p7GC) assay, NSPCs were plated in 24-well plates and were transduced with the p7GC lentiviral vector, produced as described in Supplemental experimental procedures. Next, NSPCs were washed and grown in proliferation or quiescence medium for 4 DIV. Gene expression of the eGFP WNT reporter gene was normalised to mCherry expression by RT-qPCR or alternatively, eGFP+ and mCherry+ cells were counted in a fluorescence inverted microscope LEICA DM IL LED with a 20X objective and images were acquired. For the TOPFlash Luciferase assay, Neuro2A cells were co-

transfected with M50 Super 8x TOPFlash (plasmid #12456, Addgene), Renilla luciferase, pEGFP-Lef1FL and/or pEGFP-Lef1ΔE6 (Jesse et al. 2010) plasmids using Polyethylenimine (PEI, 1mg/mL). EGFP (plasmid #6084-1, Clontech) and beta-catenin-CA plasmids were used as a control and co-factor respectively. Firefly and Renilla luciferase activity was measured using the Dual-Luciferase Reporter Assay Kit (Promega) at 48 hr post-transfection.

Motif enrichment in enhancers

DNA sequences for previously reported NSPC enhancers (Martynoga et al., 2013), with 2.5kb flanking regions, were retrieved from the masked version of the Mus musculus MGSCv37 (mm9) genome assembly. Raw count matrixes for LEF (Jaspar IDs MA0768.1, PB0040.1, PB0144.1, PF0013.1, PF0073.1), SOX2 (MA0143.3), NFIX (MA0671.1), ASCL1 (MA1100.1) and ASCL2 (MA0816.1) were downloaded from the JASPAR database (Fornes et al., 2020) and transformed into position weight matrixes (PWMs) using the toPWM function from the TFBSTools R package (Tan et al., 2016), with nucleotide frequency in the input sequences as background. PWMs were aligned to masked sequences using the matchPWM function from the Biostrings package (Tan & Lenhard, 2016; Wasserman & Sandelin, 2004), using minimal thresholds ranging from 70 to 95. Only the maximally scoring motif for each sequence was considered.

To test for enrichment in enhancers versus flanking sequences, bootstrap confidence intervals of the ratio between motifs per kb inside the enhancers and in the 2.5kb flanking regions were calculated for each enhancer group separately. In each bootstrap replicate, the location of motifs with a score of 80% or higher was randomized taking into account spatial constraints due to masked and border regions. Out of the randomized motifs, one was selected at random per enhancer, since we were only considering the highest-scoring motif in each sequence, and the length-adjusted ratio between motifs inside enhancers and in the flanking regions was calculated for each replicate. A motif is considered to be enriched in enhancers when the observed inside/outside ratio falls above the 0.975 quantile of the simulated ratios. 100 bootstrap replicates were used. [Bootstrapped intervals for each motif and enhancer type can be found in Supplementary Table 6].

To test for differential enrichment of motifs between enhancer types, only motifs with the highest scoring within each enhancer were considered, and their positions were randomized 1000 times between enhancer regions of the three groups, taking into account spatial constraints. Confidence intervals for the proportion of motifs falling in each group were calculated, and a motif was considered to be differentially enriched in an enhancer group if the proportion of motifs in the enhancer groups was above or below the confidence interval. [Bootstrapped intervals for each motif can be found in Supplementary Table 6].

In order to test for preferential LEF motif localization, we used the CentriMo tool of the MEME suite (Timothy, Bailey & Machanick, 2012), using default parameters. To search for motifs enriched in the vicinity of LEF putative binding sites, we retrieved the sequences of the LEF1 motif matches plus 15 flanking base pairs and used them as input for the DREME tool of the MEME suite (Timothy & Bailey, 2011). Enriched motifs were then fed to the TOMTOM program (Gupta et al., 2007) in order to find matches between enriched motifs and known PWMs.

Motif presence in genes differentially regulated in quiescent vs proliferative cells

We used RNA-seq and ChIP-seq data from (Martynoga et al., 2013) to test whether LEF1 motif was overrepresented in genes that were differentially regulated between quiescent and proliferative states. We considered that genes were up or down regulated if they had a p-value < 0.01 in (Martynoga et al., 2013) Supplementary Table 1. Gene-enhancer associations were taken from (Martynoga et al., 2013) Supplementary Table 2. Briefly, each gene is assigned the closest enhancer and significance of the association is assessed through a permutation test. By crossing these data with our previous motif search, we built a 3x2 contingency matrix with up-regulated, down-regulated and not differentially regulated genes vs presence and absence of LEF1 motif (Jaspar PF0013.1, score greater or equal to 85%) and used Chi-squared test.

References

Bailey, T.L. and Machanick, P. "Inferring direct DNA binding from ChIP-seq", Nucleic Acids Research, 40:e128, 2012.

Bailey, T.L. "DREME: Motif discovery in transcription factor ChIP-seq data", Bioinformatics, 27(12):1653-1659, 2011.

Fornes, O., Castro-Mondragon, J.A., Khan. A., et al. JASPAR 2020: update of the open-access database of transcription factor binding profiles. Nucleic Acids Res. 2019; doi: 10.1093/nar/gkz1001.

Frazee, A. C. et al. (2015). Ballgown bridges the gap between transcriptome assembly and expression analysis. Nat. Biotechnol. 33, 243–246

Gupta, S., Stamatoyannopolous, J.A., Bailey, T.L. and Stafford Noble, W. "Quantifying similarity between motifs", Genome Biology, 8(2):R24, 2007.

Jesse, S., Koenig, A., Ellenrieder, V. and Menke, A. (2010). Lef-1 isoforms regulate different target genes and reduce cellular adhesion. Int. J. Cancer: 126, 1109–1120

Kim, D., Langmead, B. & Salzberg, S. L. (2015). HISAT: a fast spliced aligner with low memory requirements. Nat. Methods 12, 357–360

Li, C. and Wong, W.H. (2001). Model-based analysis of oligonucleotide arrays: expression index computation and outlier detection. Proc.Natl.Acad.Sci.U.S.A;98(1)31-36

Livak, K.J., and Schmittgen, T.D. (2001). Analysis of relative gene expression data using real-time quantitative PCR and the 2(-Delta Delta C(T)) Method. Methods 25, 402–408.

Mira, H., Andreu, Z., Suh, H., Lie, D.C., Jessberger, S., Consiglio, A., San Emeterio, J., Hortigüela, R., Marqués-Torrejón, M.A., Nakashima, K., et al. (2010). Signaling through BMPR-IA regulates quiescence and long-term activity of neural stem cells in the adult hippocampus. Cell Stem Cell 7, 78–89.

Pagès, H., Aboyoun, P., Gentleman, R., DebRoy, S. (2021). Biostrings: Efficient manipulation of biological strings. R package version 2.60.2, https://bioconductor.org/packages/Biostrings.

Palmer, T. D., Takahashi, J. and Gage, F. H. (1997). The adult rat hippocampus contains primordial neural stem cells. Mol. Cell. Neurosci. 8, 389–-404

Pertea, M. et al. (2015). StringTie enables improved reconstruction of a transcriptome from RNA-seq reads. Nat. Biotechnol. 33, 290–295

Tan, G., Lenhard, B. (2016). TFBSTools: an R/Bioconductor package for transcription factor binding site analysis. Bioinformatics, 32, 1555-1556.

Wasserman, WW, Sandelin, A., (2004). Applied bioinformatics for the identification of regulatory elements, Nat Rev Genet., 5(4):276-87.

Supplementary Figure 1

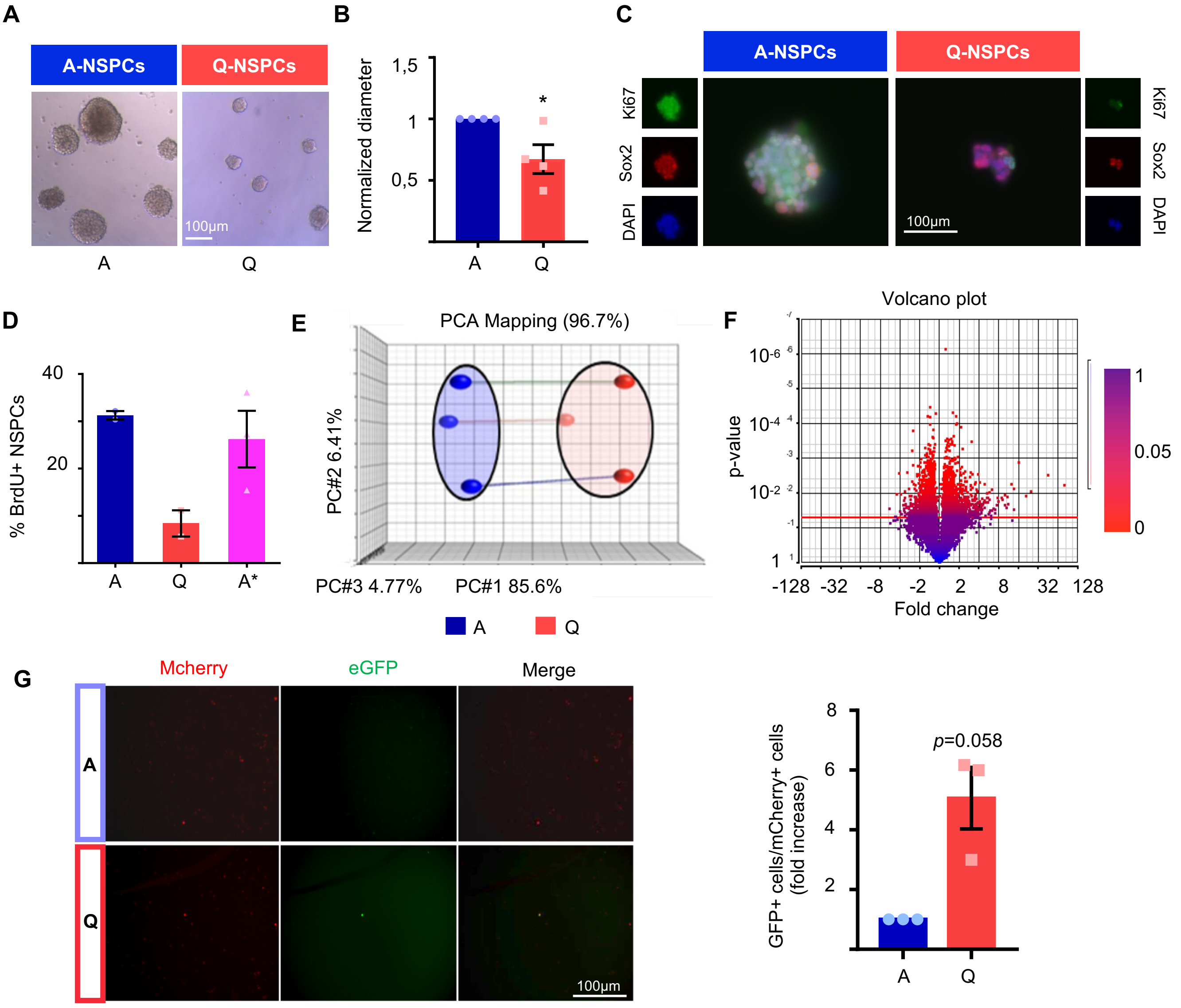

Supplementary Figure 1. (A) Brightfield images of active and quiescent NSPC neurospheres at 40X showing the decreased size of the quiescent neurosphere clonal aggregates. **(B)** Normalized quantification of the diameter of the neurospheres from Q-NSPCs *vs.* A-NSPCs (n=4, T-test, $p<0.05$). **(C)** Confocal images of Sox2 (red) and Ki67 (green) immunostained cells in active and quiescent NSPC neurospheres. Nuclear staining (DAPI) is shown in blue. **(D)** Quantification of BrdU incorporation in Active (A) 4 DIV, Quiescent (Q) 4 DIV and reactivated NSPCs in the presence of Noggin (A*) 3DIV (n≥2). **(E)** Principal component analysis (PCA) plot of the A- and Q-NSPCs samples using the significant gene expression list obtained from the genome-wide microarray analysis. Samples are coloured by condition (blue, A-NSPCs and red, Q-NSPCs) and connected by replicate Match. Percentage of variability explained by the model: 85.6% (axis 1); 6.41% (axis 2); 4.77% (axis 3). **(F)** Volcano plot with included *p*-values showing the comparison of Q-NSPCs *vs.* A-NSPCs. Each point represents one of the probe sets of the working list. Probes are coloured according the signification obtained as shown in the legend. **(G)** Representative image *(left)* and fold increase *(right)* in GFP+ and mCherry+ cells transfected with lentiviral 7xTCF/LEF1-eGFP/SV40-mCherry reporter. Data are presented as Mean ± SEM. *$p<0.05$.

Supplementary Figure 2

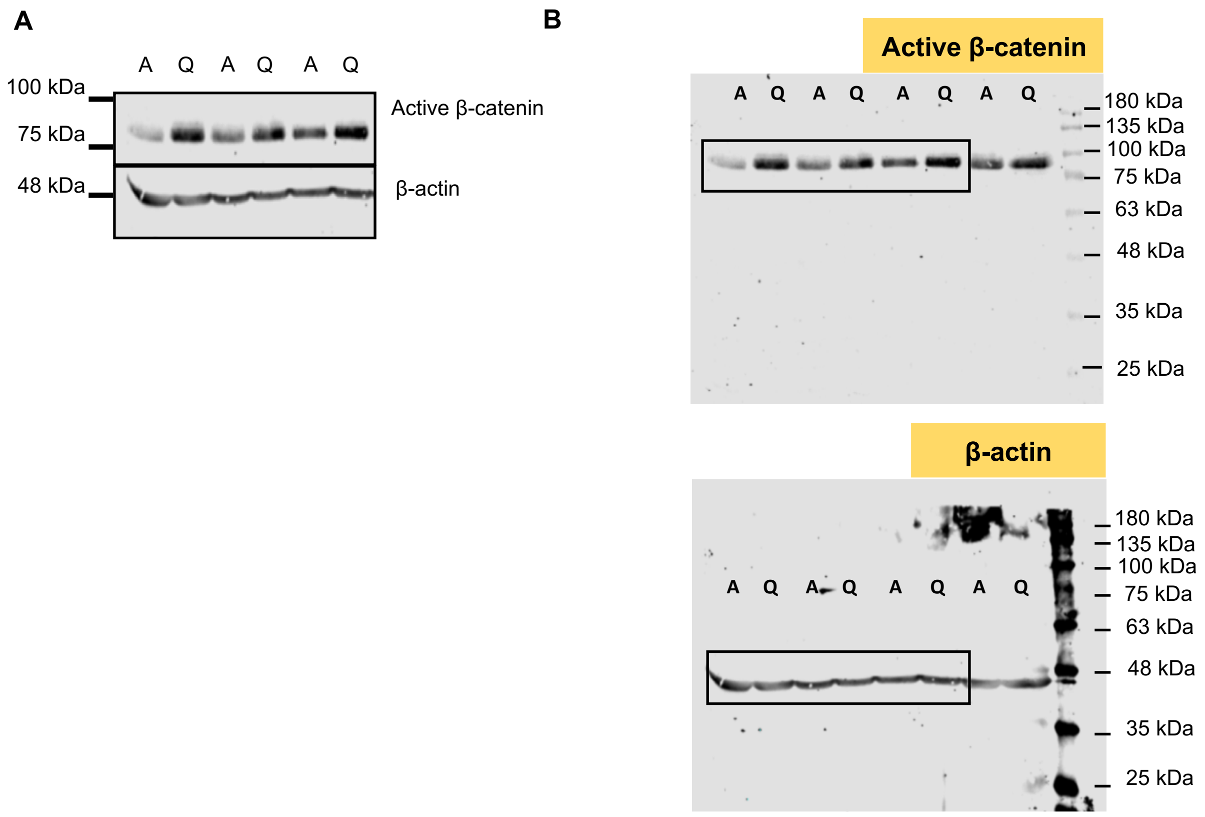

Supplementary Figure 2. (A) Western blot shown in Figure 2E. **(B)** Original full-length Western immunoblots showing the selected region used to compose the final image displayed in (A).

Supplementary Figure 3

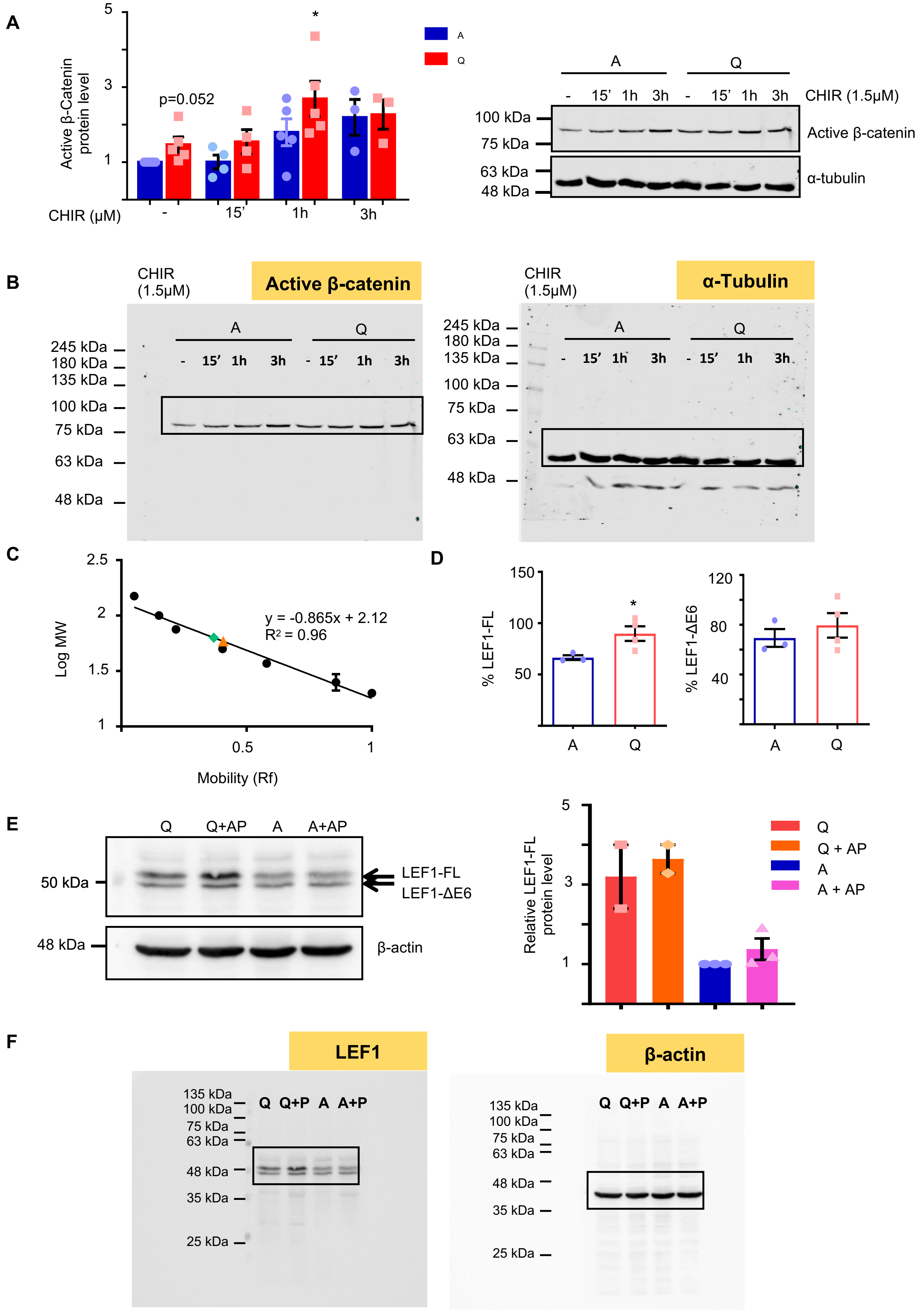

Supplementary Figure 3. (A) Representative Western blot (*right*) and quantification (*left*) of relative β-catenin levels as analyzed by Western blot in A- and Q-NSPCs treated with 1.5μM CHIR99021 (abbreviated as CHIR) at different timepoints (n≥ 3, one-way ANOVA, *p<0.05). Additional statistical analysis by T-test is also provided for untreated A- and Q-NSPCs. α-Tubulin was used as loading control. **(B)** Original full-length Western immunoblots showing the selected region used to compose the final image displayed (A). **(C)** Theoretical calculation of the protein weight difference between the LEF1 upper band (green symbol) and lower band (orange symbol) as estimated from the linear regression curve corresponding to the LogMW (molecular weight, kDa) *vs.* Relative migration distance (Rf) of protein standards measured by SDS-PAGE mobility. The two LEF1 protein bands differed in about 4.35±0.33 kDa. **(D)** Half-life ($t_{1/2}$) assays were used to analyze the stability of LEF1 upper and lower band (n≥3), T-test, p<0.05). **(E)** Representative Western blot (*left*) and relative LEF1-FL protein levels (*right*) in Q- and A-NSPCs treated with Alkaline Phosphatase (AP). The data show that the mobility of the LEF1 band was unrelated to Ser phosphorylation (n≥2). β-actin was used as loading control. **(F)** Original full-length Western immunoblots showing the selected region used to compose the final image displayed in (E). Data are presented as Mean ± SEM. *p<0.05; **p<0.01; ***p<0.001.

Supplementary Figure 4

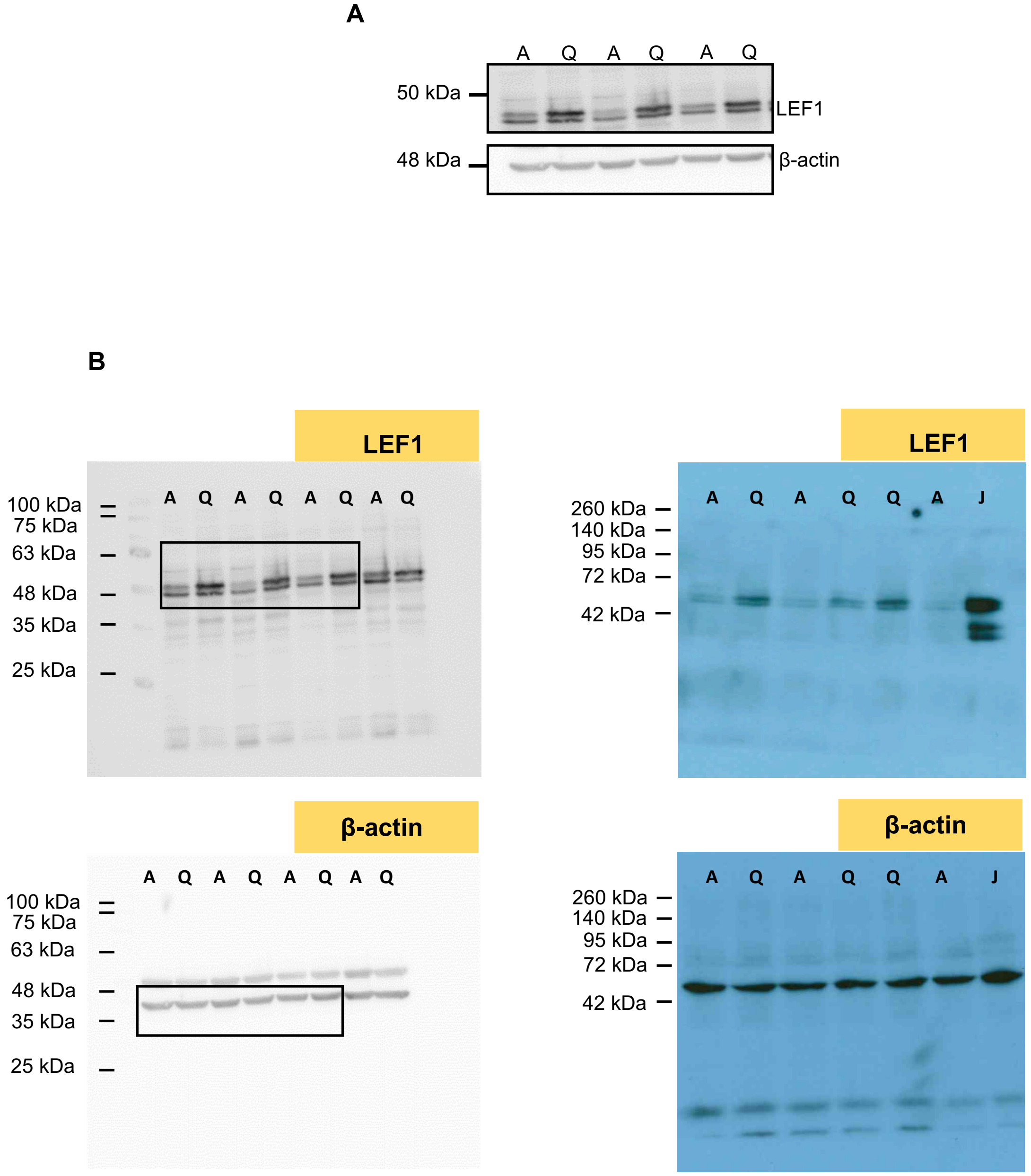

Supplementary Figure 4. (A) Western blot shown in Figure 2I. **(B)** Original full-length Western immunoblots. The *left panel* showing the selected region used to compose the final image displayed in (A). In the *right panel*, J corresponds to a protein extract from the human leukaemic T cell lymphoblast cell line Jurkat, used as a positive control for the antibody.

Supplementary Figure 5

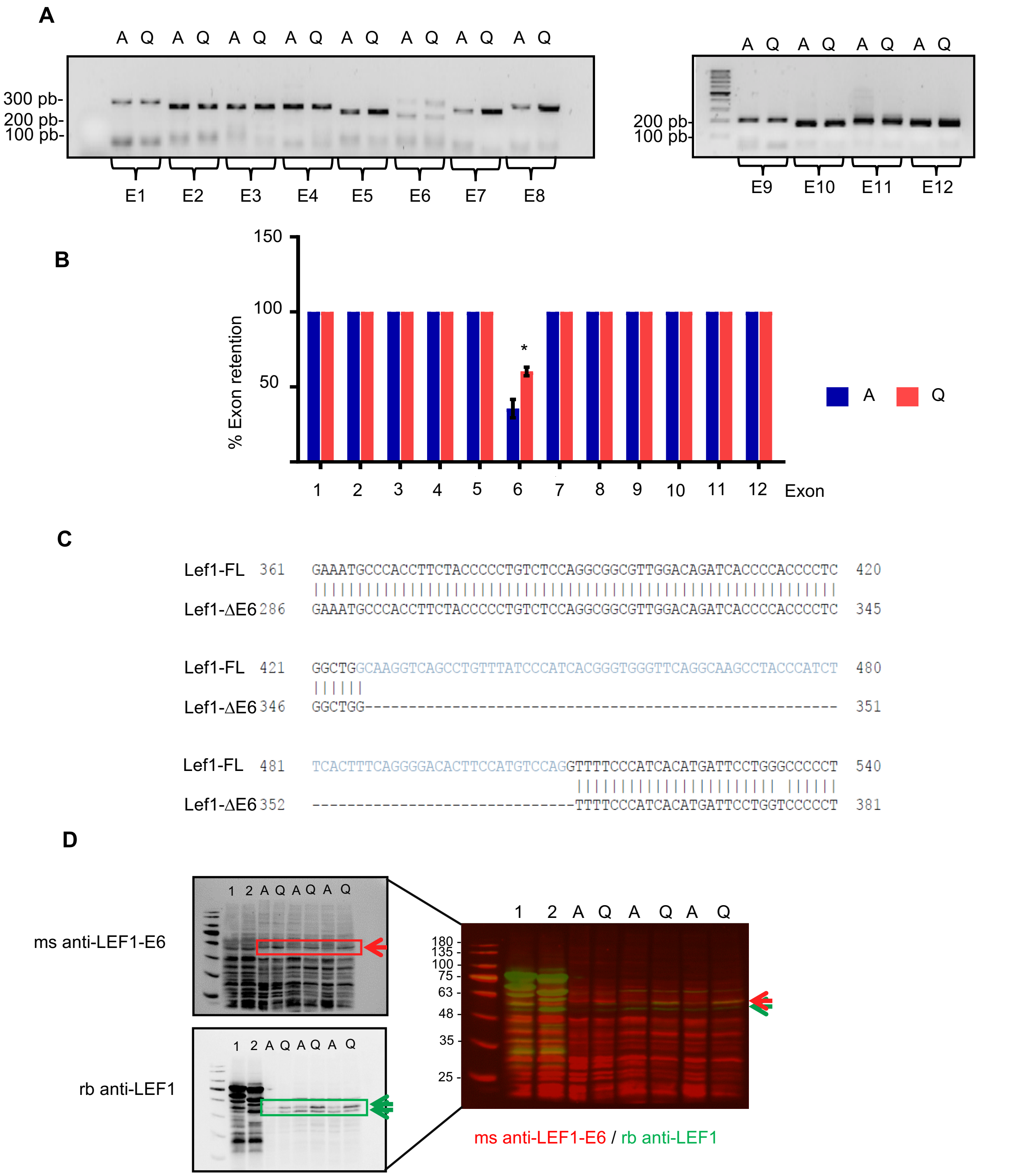

Supplementary Figure 5. (A) Representative example of the *Lef1* PCR employing primer pairs flanking all *Lef1* exons (E1 to E12). cDNA was obtained from A- and Q-NSPCs and PCR products were resolved by agarose gel electrophoresis. **(B)** Percentage of exon retention in Q-NSPCs *vs*. A-NSPCs (n=3, T-test, p<0.01). Note that only E6 underwent alternative splicing in NSPCs and was retained in the quiescent state. **(C)** Sequencing of inferior band of cDNA from A-NSPCs employing primer pairs flanking exon 6 showing the alternative spliced amplicon of *Lef1*. **(D)** *Left panel*, Western blot analysis of A- and Q-NSPCs protein extracts (3 independent experiments) employing a rabbit anti-LEF1 antibody that recognizes all isoforms and a mouse anti-LEF1-E6 antibody raised against a conserved epitope mapping the CRD residues encoded by E6 (lane 1 and 2 are unrelated HEK293 cell extracts), therefore recognizing the LEF1-FL but not the LEF1-ΔE6 isoform. *Right panel*, overlay of the signals.

Supplementary Figure 6

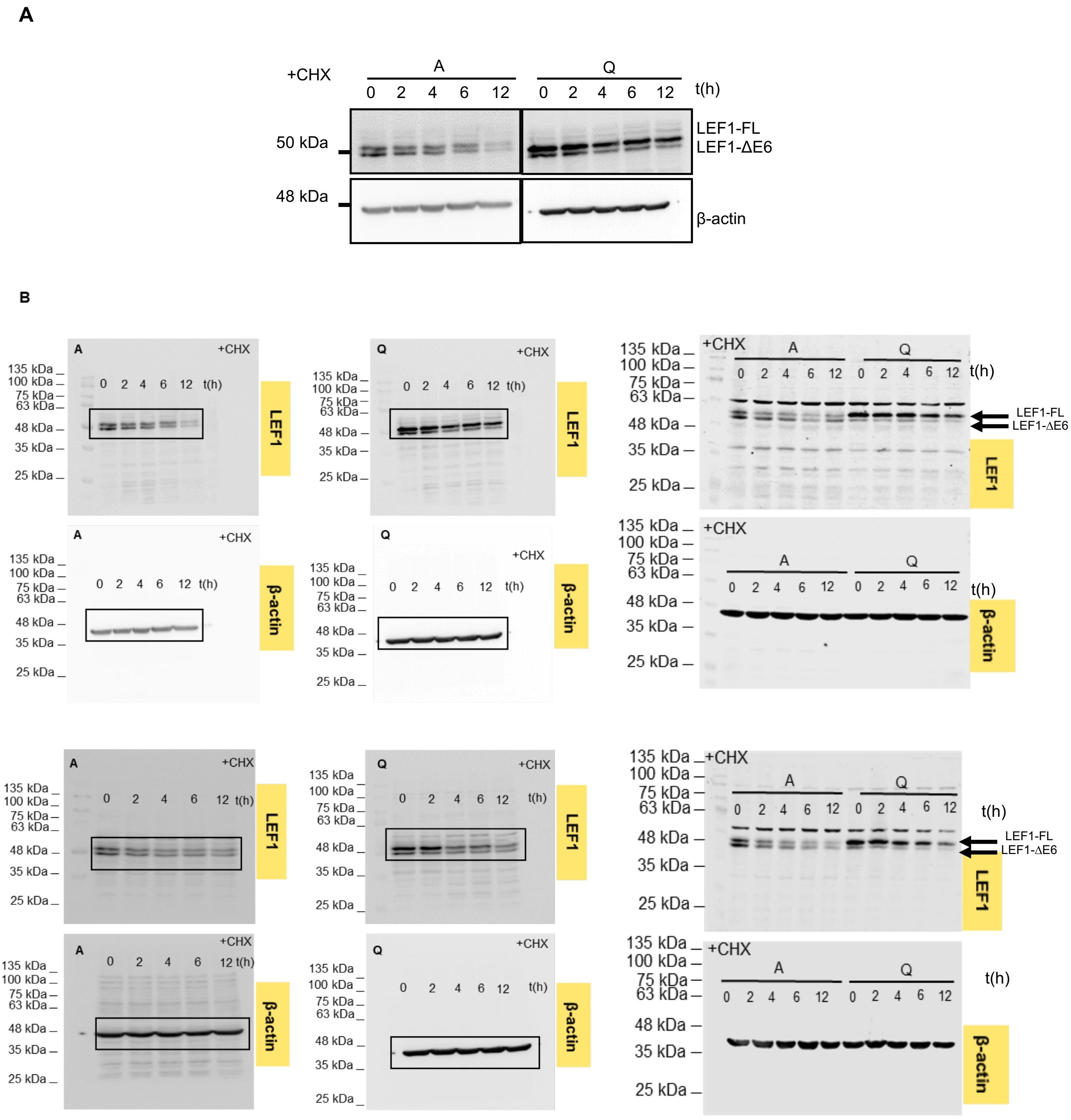

Supplementary Figure 6. (A) Western blot shown in Figure 3G. **(B)** Original full-length Western immunoblots from independent experiments showing the selected region used to compose the final image displayed in (A) and to quantify the protein half-life as shown in figure 3H.

Supplementary Figure 7

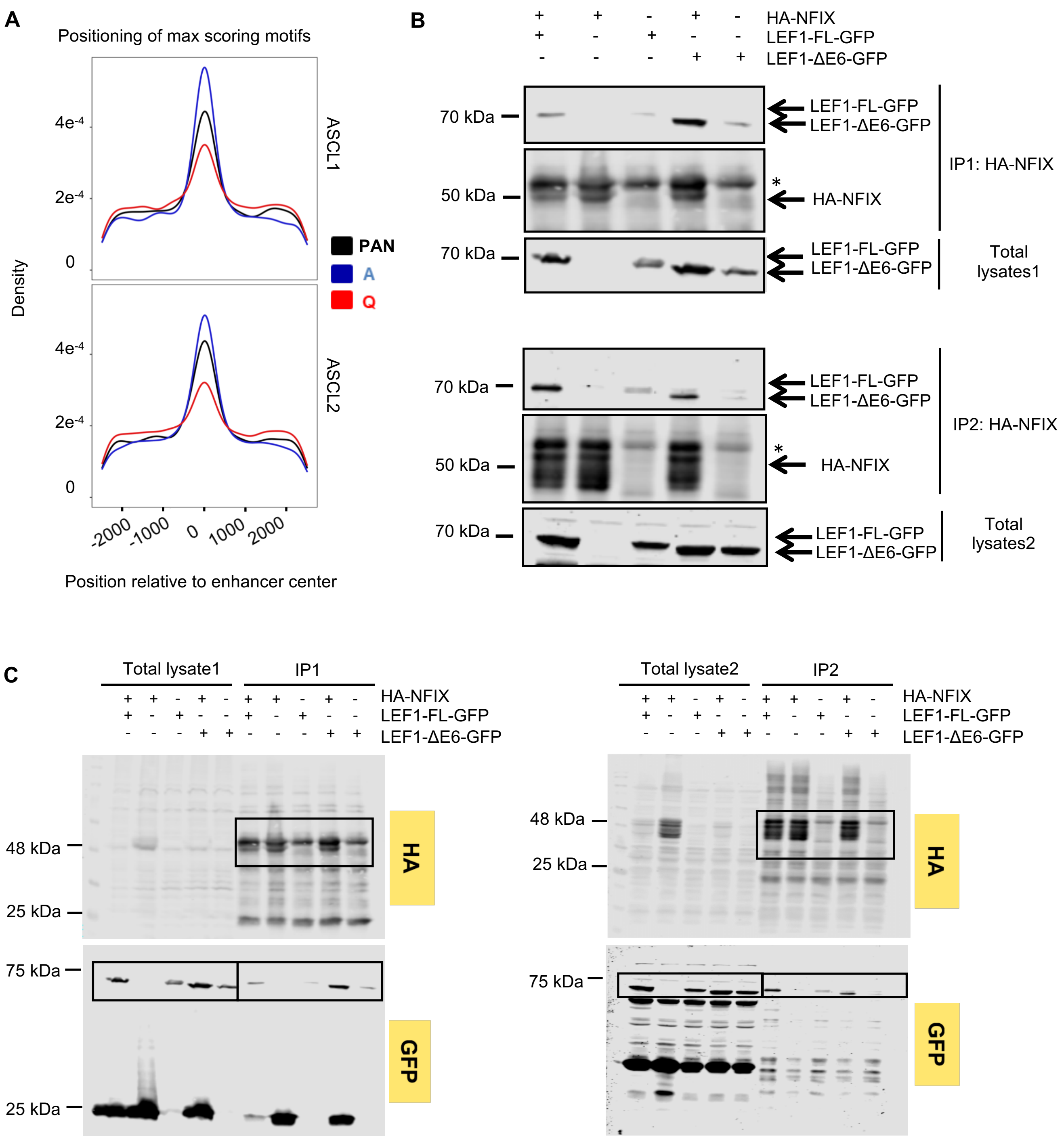

Supplementary Figure 7. (A) Enrichment of the ASCL1 and ASCL2 motifs in A-NSPC, Q-NSPC and PAN enhancer groups. **(B)** Co-immunoprecipitation and Western blot analysis of the HA-tagged NFIX and GFP-tagged LEF1-FL or LEF1-ΔE6 isoforms expressed in HEK293 cells, showing the NFIX/LEF1 interaction in both isoforms. The Co-IP was performed in n=3 independent experiments. The blots correspond to two of them. **(C)** Original full-length Western immunoblots from 2 independent experiments showing the selected region used to compose the final image displayed (B). Data are presented as Mean ± SEM.

Supplementary Figure 8

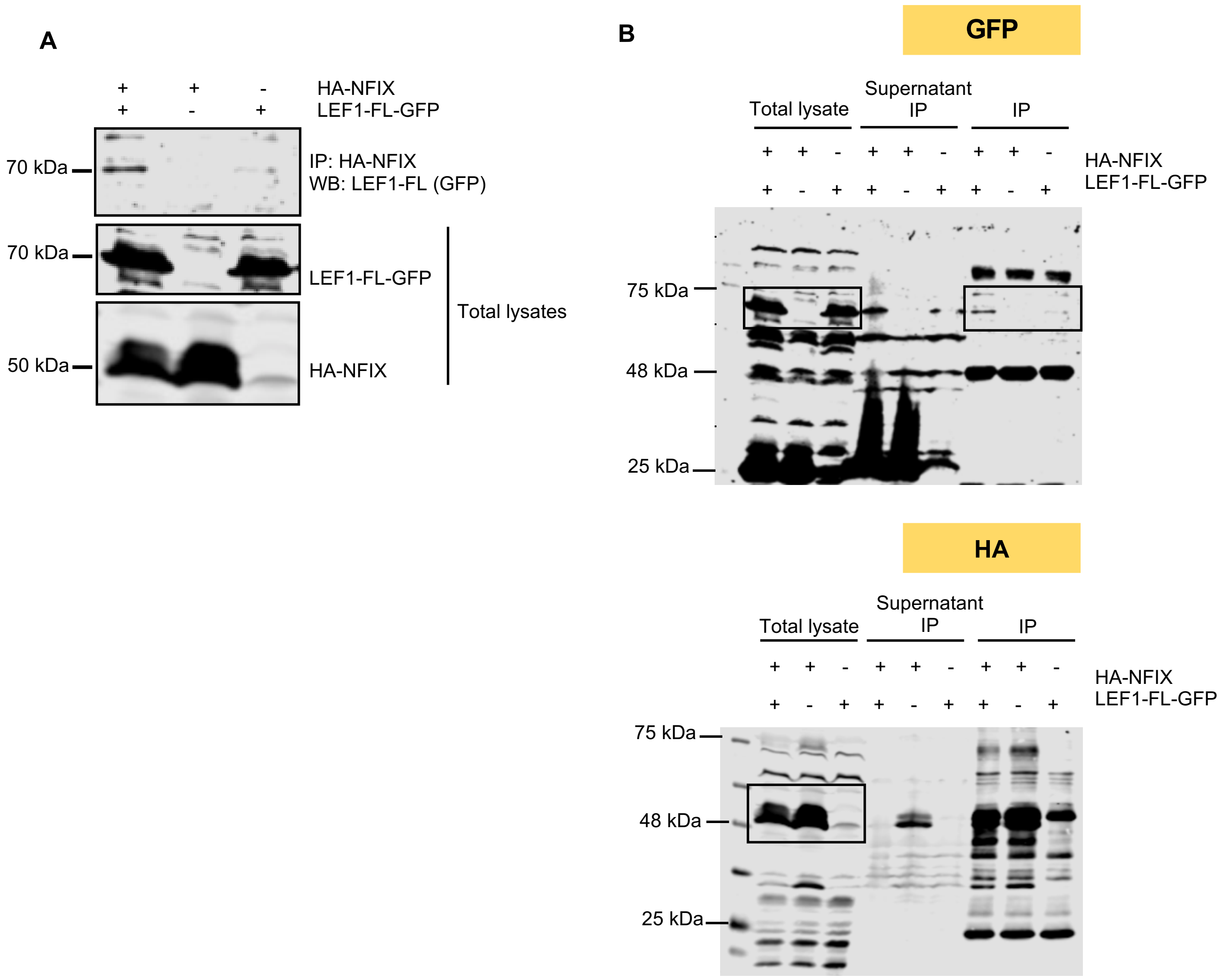

Supplementary Figure 8. (A) Western blot analysis of co-immunoprecipitation shown in Figure 4E. **(B-C)** Original full-length Western immunoblots showing the selected region used to compose the final image displayed in (A).

Supplementary Figure 9

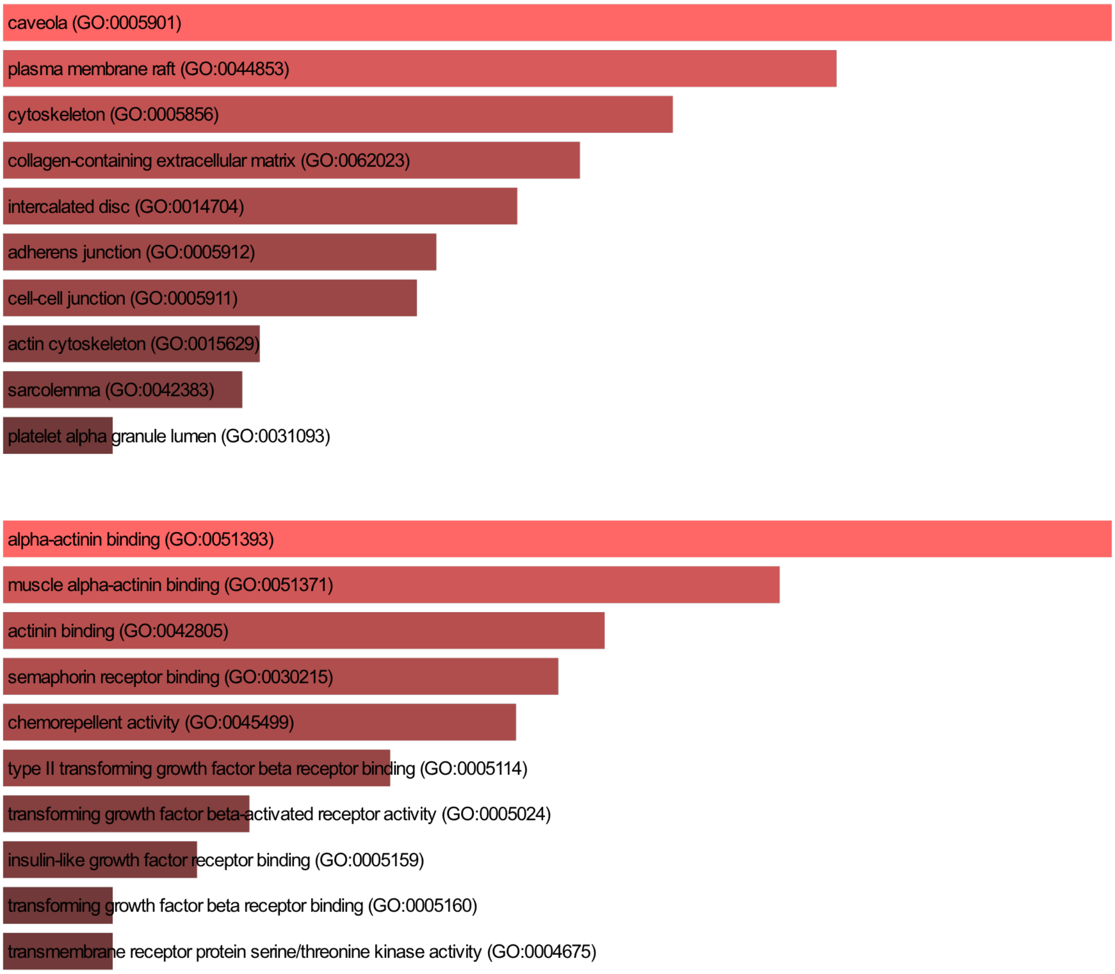

Supplementary Figure 9. Functional enrichment with Gene Ontology (GO) showed that mRNAs up-regulated in Q-NSPCs having active enhancers with a LEF1 site were mostly involved in plasma membrane and extracellular processes, such as plasma membrane rafts, cell-cell junctions or extracellular matrix, as well as in TGF-beta and Insulin-like growth factor receptor signalling.
